# SARS-CoV-2 nucleocapsid protein forms condensates with viral genomic RNA

**DOI:** 10.1371/journal.pbio.3001425

**Published:** 2021-10-11

**Authors:** Amanda Jack, Luke S. Ferro, Michael J. Trnka, Eddie Wehri, Amrut Nadgir, Xammy Huu Wrynla, Douglas Fox, Katelyn Costa, Sarah Stanley, Julia Schaletzky, Ahmet Yildiz

**Affiliations:** 1 Biophysics Graduate Group, University of California, Berkeley, California, United States of America; 2 Department of Molecular and Cell Biology, University of California, Berkeley, California, United States of America; 3 Department of Pharmaceutical Chemistry, University of California, San Francisco, California, United States of America; 4 Center for Emerging and Neglected Diseases, University of California, Berkeley, California, United States of America; 5 Physics Department, University of California, Berkeley, California, United States of America; 6 School of Public Health, Division of Infectious Diseases and Vaccinology, University of California, Berkeley, California, United States of America; 7 Press West Illustrations, Boston, Massachusetts, United States of America; Centre International de Recherche en Infectiologie (CIRI), FRANCE

## Abstract

The Severe Acute Respiratory Syndrome Coronavirus 2 (SARS-CoV-2) infection causes Coronavirus Disease 2019 (COVID-19), a pandemic that seriously threatens global health. SARS-CoV-2 propagates by packaging its RNA genome into membrane enclosures in host cells. The packaging of the viral genome into the nascent virion is mediated by the nucleocapsid (N) protein, but the underlying mechanism remains unclear. Here, we show that the N protein forms biomolecular condensates with viral genomic RNA both in vitro and in mammalian cells. While the N protein forms spherical assemblies with homopolymeric RNA substrates that do not form base pairing interactions, it forms asymmetric condensates with viral RNA strands. Cross-linking mass spectrometry (CLMS) identified a region that drives interactions between N proteins in condensates, and deletion of this region disrupts phase separation. We also identified small molecules that alter the size and shape of N protein condensates and inhibit the proliferation of SARS-CoV-2 in infected cells. These results suggest that the N protein may utilize biomolecular condensation to package the SARS-CoV-2 RNA genome into a viral particle.

## Abstract 2

The packaging of the SARS-CoV-2 genome is mediated by the nucleocapsid (N) protein; this study shows that the N protein forms liquid condensates with viral genomic RNA and identifies small molecules that alter these condensates.

## Introduction

The Severe Acute Respiratory Syndrome Coronavirus 2 (SARS-CoV-2) virus consists of a 30-kb single-stranded RNA genome packaged into a 100-nm diameter membrane enveloped virion. SARS-CoV-2 encodes for multiple proteins involved in viral assembly and propagation [1] and infects human cells by binding its spike (S) protein to the angiotensin converting enzyme 2 (ACE2) receptor on host cells [2–4]. While the majority of current efforts to treat Coronavirus Disease 2019 (COVID-19) have focused on targeting this interaction [5,6], not much work has been done to stop the proliferation of the virus in host cells following infection. Condensation of the viral genome into a virion is primarily driven by the nucleocapsid (N) protein [7], which is the most abundant viral protein in infected cells [8,9]. A large pool of free N protein is expressed early in infection [10], and only a small fraction is transferred into mature virions [9]. The N protein accumulates at the replication transcription complex (RTC) [11,12] where it enhances replication and the transcription of viral RNA [13,14]. The N protein also restructures viral genomic RNA into shell-shaped structures (approximately 15 nm in diameter), which contain approximately 12 N proteins and 800 nucleotides of viral RNA [7,15]. These viral ribonucleoprotein (vRNP) complexes form asymmetric “beads on a string” structures that then bind to the viral membrane (M) protein on the surface of the ER–Golgi intermediate compartment (ERGIC) to trigger the budding of the vRNP complex.

The mechanism by which N remodels the viral RNA and packages it into a viral particle is not well understood. Recent studies proposed that the replication and packaging of viruses involve liquid–liquid phase separation (LLPS) [16–18]. Biomolecular condensation drives the formation of membrane-less organelles such as the nucleolus, centrosome, stress granules, and P granules through a network of weak and multivalent interactions. Nucleic acids are involved in the formation of biomolecular condensates because they can scaffold multivalent interactions [19–21]. Coronaviruses are involved with phase-separated structures such as stress granules [1,10] and replicate at dynamic clusters associated with the ERGIC, suggesting that phase separation may play a critical role in the replication and packaging of SARS-CoV-2.

**Fig 1:**
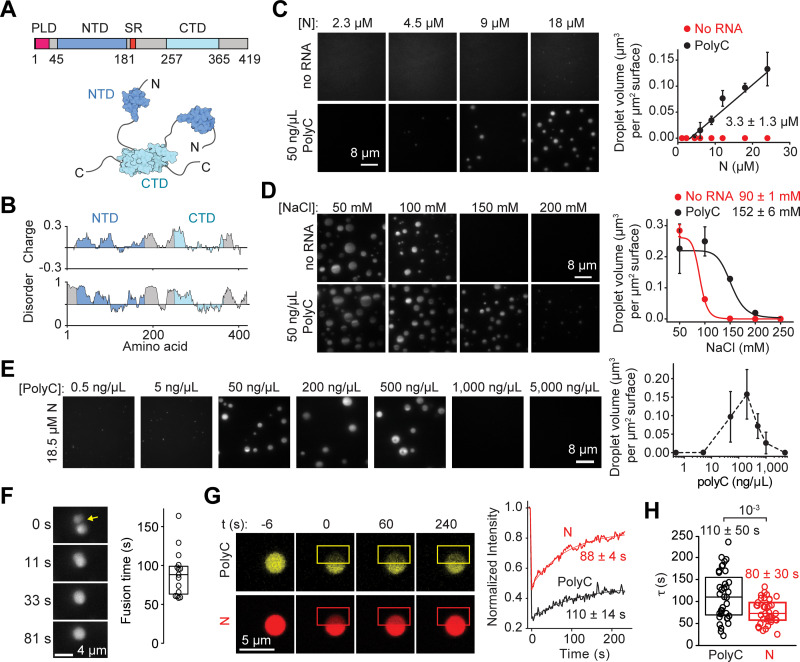
The SARS-CoV-2 N protein phase separates with RNA in vitro. **(A)** Domain organization and the schematic of the N protein dimer. **(B)** Sliding window plot of charge distribution (EMBOSS) and disorder prediction (IUPred2A) for the N protein. Charge y-axis represents mean charge across a 30-residue sliding window. Disorder prediction (1, disordered; 0, ordered) was calculated using the “long disorder” setting, encompassing a 30-residue sliding window. **(C)** (Left) Images of the LD655-labeled N protein in the presence and absence of polyC RNA in 150 mM NaCl. (Right) The total volume of N-RNA condensates settled per micron squared area on the coverslip with and without 50 ng/μL polyC RNA (mean ± SD, *n* = 20 with 2 technical replicates). A linear fit (solid line) reveals c_sat_ (± SE), the minimum N protein concentration for condensate formation (see Methods). **(D)** (Left) Condensates formed by 24 μM LD655-labeled N protein in the presence or absence of 50 ng/μL polyC RNA dissolve by increasing NaCl concentration. (Right) The total volume of N condensates settled per micron squared area on the coverslip with increasing salt concentration (mean ± SD, *n* = 10). Solid curves represent a fit to a dose–response equation to determine IC_50_ (± SE). **(E)** The stoichiometry of the N protein and RNA affects phase separation. (Left) Example pictures show that Cy3-labeled N protein forms condensates with different concentrations of polyC RNA. The N protein concentration was set to 18.5 μM. (Right) The total volume of N-polyC condensates settled per micron squared area on the coverslip under different RNA concentrations (mean ± SD; *n* = 20, 2 technical replicates). **(F)** (Left) The fusion of N-polyC condensates formed in the presence of 18.5 μM LD655-labeled N and 50 ng/μL polyC RNA. (Right) Fusion time of N-polyC condensates (mean ± SD, *n* = 15 fusion events). The center and edges of the box represent the median with the first and third quartiles. **(G)** (Left) Representative FRAP imaging of an N-polyC condensate. The image of a condensate before the time of photobleaching (0 second) shows colocalization of Cy3-polyC and LD655-N in the condensate. Rectangles show the photobleached area. (Right) Fluorescence recovery signals of N and polyC in the bleached region. Solid curves represent a single exponential fit to reveal the recovery lifetime (τ, ±95% confidence interval). **(H)** The distribution of fluorescence recovery lifetimes of N and polyC in droplets (*n* = 37). The center and edges of the box represent the median with the first and third quartiles. The *p*-value was calculated from a 2-tailed *t* test. Data underlying this figure can be found in S1 Data. CTD, carboxyl-terminal domain; N, nucleocapsid; NTD, N-terminal domain; PLD, prion-like domain; SARS-CoV-2, Severe Acute Respiratory Syndrome Coronavirus 2; SR, serine/arginine-rich.

Previous studies in positive and negative-sense RNA viruses showed that N proteins drive the formation of phase-separated condensates in the cytosol [22–25]. The N protein of SARS-CoV-2 also contains many of the characteristic domain features common in phase separating proteins. It contains a well-conserved N-terminal domain (NTD) and a carboxyl-terminal domain (CTD, Fig 1A), and 40% of its primary sequence is predicted to be part of intrinsically disordered regions (IDRs, Fig 1B). The NTD (amino acids 44 to 174) interacts nonspecifically with RNA and recognizes a nucleotide sequence in the 3′ end of the viral genome [13]. The CTD (amino acids 257 to 366) mediates dimerization [26], but the N protein can also self-associate into tetramers and higher oligomers [27,28]. The NTD and CTD are separated by an IDR that contains a serine/arginine-rich (SR) motif, which has been associated with phase separation in other ribonucleoproteins [13,29]. The N and carboxyl-terminal IDRs are less conserved but contain arginine- and lysine-rich disordered regions (Fig 1B), which may facilitate additional interactions with the negatively charged RNA backbone [30] and drive biomolecular condensation of RNA [31,32]. The N-terminal IDR contains a predicted prion-like domain (PLD, Fig 1A) that can potentially trigger protein demixing [13,29,33]. The carboxyl-terminal IDR of SARS-CoV N mediates binding to the M protein [34,35]. The N protein is highly positively charged (+24 in pH 7.4) [36], and the CTD and disordered regions also interact with negatively charged RNA and promote vRNP packaging [37]. The precise roles of these domains in the phase separation of N protein remain to be elucidated.

In this study, we purified the SARS-CoV-2 N protein from human embryonic kidney (HEK293) cells and observed that N protein forms biomolecular condensates with both homopolymeric and viral genomic RNA under physiological salt conditions in vitro. We also showed that the N protein forms liquid condensates in mammalian cells. Cross-linking mass spectrometry (CLMS) identified 2 regions flanking the CTD with interactions enriched within the condensed phase, and the deletion of one of these regions fully abrogated condensate formation in vitro. Together, our results indicate that the N protein phase separates with genomic RNA of SARS-CoV-2, which may play an important role in the packaging of new viral particles in host cells.

## Results

### The N protein phase separates with RNA

We first asked whether the N protein forms bimolecular condensates in the presence or absence of viral RNA in vitro. To address this, we expressed wild-type (WT) N protein in HEK293 cells and purified it in a high salt buffer (1 M NaCl) to eliminate the retention of RNA from human cells [38] (S1A and S1B Fig). Consistent with recent studies, purified N protein eluted from gel filtration as an oligomer in 300 mM NaCl [26] and had a high affinity for binding to viral and nonviral RNA substrates [39,40] (S1C–S1F Fig). The protein was labeled with a fluorescent dye (LD655) at the carboxyl-terminal ybbR tag and introduced to a flow chamber in the presence or absence of RNA. Phase separation was monitored by the settling of N or N-RNA condensates onto the coverslip within 25 minutes under highly inclined and laminated optical sheet (HiLO) excitation (see Methods for details). In the absence of RNA, N protein did not form condensates in physiological salt (150 mM NaCl) (Fig 1C). Similarly, 2-kb long polyC RNA homopolymer did not form any condensates in the absence of N protein (S2A and S2B Fig). Mixing of 50 ng/μL polyC RNA and the N protein resulted in the formation of condensates in 150 mM NaCl (Fig 1C). LD655-N and Cy3-polyC colocalized in the droplets, suggesting that phase separation of N protein is mediated by RNA (S2C Fig). The analysis of the condensates settled on the coverslip revealed a saturation concentration (c_sat_) of 3.3 ± 1.3 μM for N protein in the presence of 50 ng/μL polyC RNA (± SE, Fig 1C), lower than the concentration of N protein in infected cells [41]. The partition coefficient of N protein into condensates was 13 ± 2 (± SD).

Although N protein is unable to phase separate in the absence of RNA in 150 mM salt, 24 μM N protein efficiently formed condensates without RNA at lower salt with a half-maximal inhibition constant (IC_50_) of 90 ± 1 μM NaCl (± SE) (Fig 1D). The addition of 50 ng/μL polyC RNA increased IC_50_ to 152 ± 6 mM NaCl (Fig 1D). The ability of N protein to phase separate without RNA and sensitivity of these condensates to salt indicates that these condensates may be driven, in part, by electrostatic interactions among N proteins, as observed for other phase separating systems [42–45].

Condensate formation of N protein and RNA was dependent on protein–RNA stoichiometry. At 18 μM N protein, condensate formation was not observed in the presence of 0 to 5 ng/μL polyC RNA. Increasing the RNA concentration promoted phase separation with an optimal RNA concentration of 100 to 500 ng/μL, at which charge neutralization occurs with the positively charged N protein [46]. A further increase in RNA concentration dissolved these condensates (Fig 1E) [47,48]. This reentrant behavior is characteristic of heterotypic RNA and protein interactions in phase separating systems [47].

We also showed that N-polyC condensates exhibit liquid-like, rather than solid-like, material properties. First, these condensates were nearly spherical with an aspect ratio of 1.3 ± 0.6 (mean ± SD). Second, we observed the fusion of condensates with a mean fusion time of 90 ± 30 seconds (mean ± SD) after they come into contact (Fig 1F). Finally, fluorescence recovery after photobleaching (FRAP) experiments showed that 90 ± 10% of N protein in condensates can slowly exchange with the solvent with the half recovery time of 80 ± 30 seconds (mean ± SD). In comparison, polyC RNA exhibited slower recovery and a lower mobile fraction (Fig 1G and 1H, S3 Fig), indicating that the RNA may be stabilized by a network of interactions with multiple N proteins in the condensates.

**Fig 2:**
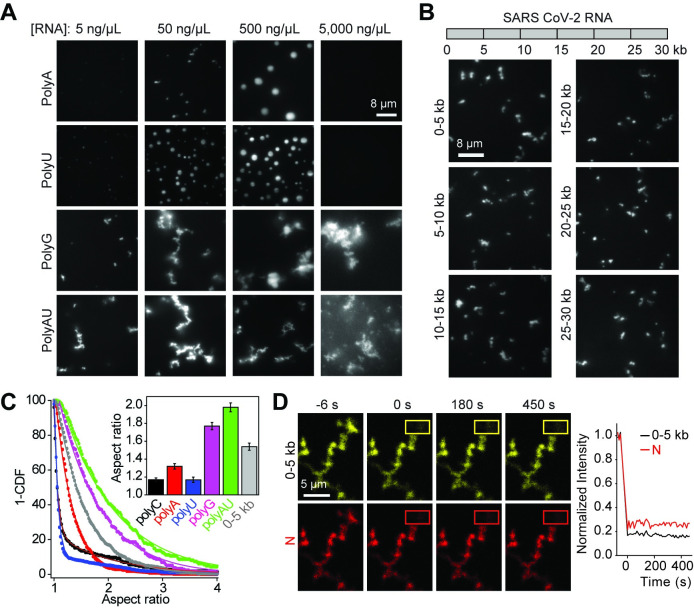
The N protein forms asymmetric condensates with viral RNA. **(A)** The N protein forms spherical condensates with polyA and polyU RNA but forms asymmetric condensates with substrates that form Hoogsteen (polyG) and Watson–Crick (polyAU) base pairing interactions. The N protein concentration was set to 18.5 μM. **(B)** (Top) SARS-CoV-2 genomic RNA was divided into six 5-kb sections. (Bottom) The formation of asymmetric N condensates in the presence of 18 nM RNA. The N protein concentration was set to 18.5 μM. **(C)** The inverse cumulative distribution (1-CDF) of the aspect ratio of individual N condensates formed with different RNA substrates. The concentrations of N protein, RNA homopolymers, and 0–5 kb viral RNA were set to 18.5 μM, 50 ng/μL, and 18 nM, respectively. Solid curves represent a fit to exponential decay. (Insert) Decay constants of the exponential fits (± SE). **(D)** (Left) Representative FRAP imaging of an N and 0–5 kb viral RNA condensate. The image of a condensate before the time of photobleaching (0 second) shows colocalization of Cy3-labeled 0–5 kb viral RNA and LD655-N in the condensate. Rectangles show the photobleached area. (Right) N and 0–5 kb viral RNA do not exhibit fluorescence recovery in the bleached region (*n* = 16). Data underlying this figure can be found in S1 Data. CDF, cumulative distribution function; N, nucleocapsid; SARS-CoV-2, Severe Acute Respiratory Syndrome Coronavirus 2.

Similar to polyC RNA, polyA and polyU RNA substrates produced spherical condensates at micromolar concentrations of the N protein (Fig 2A) [49]. However, combining N protein with polyG that forms Hoogsteen base pairing [50] or polyAU that forms Watson–Crick base pair interactions [51] led to the formation of nonspherical condensates (Fig 2A). These results indicate that the N protein forms liquid condensates with some of the homopolymeric RNA substrates, whereas other homopolymeric RNA substrates that form base pairing interactions result in asymmetric condensates [19,52–54].

Next, we sought to characterize how the N protein interacts with SARS-CoV-2 genomic RNA. The SARS-CoV-2 RNA genome was reverse transcribed and assembled into a DNA plasmid [55]. Using this plasmid, we generated six 5-kb fragments (Fig 2B) and two 1-kb fragments of the viral RNA genome via in vitro transcription [56]. In silico methods predict that these RNA fragments can form intra- and intermolecular base pairing interactions and contain extensive secondary structure elements (S4 Fig) [52,57,58]. The N protein formed nonspherical condensates with viral RNA fragments across a wide range of protein and viral RNA concentrations (Fig 2B and 2C, S4B–S4D Fig). These condensates were dissolved by increasing the salt concentration, but they neither changed shape over time, fused with each other, nor were strongly affected by raising the temperature from 20°C to 37°C (S5 Fig). We also did not detect fluorescence recovery of either the LD655-labeled N protein or Cy3-labeled viral RNA in FRAP assays (Fig 2D, S6 Fig). We concluded that N protein forms solid-like condensates with viral RNA in vitro and that base pairing interactions of the RNA may influence the material properties of the condensate [19,52,53].

### CLMS identifies N protein interaction sites

To understand the mechanism of phase separation of the N protein, we performed CLMS to identify interactions between different domains of the full-length N protein in the absence of RNA [59]. CLMS detects protein–protein contacts by covalently capturing closely positioned lysine residues with bifunctional reagents. We first cross-linked the soluble (not phase separated) N protein in 300 mM KAc using a bifunctional cross-linker bis(sulfosuccinimidyl) suberate (BS3) (S7A Fig) [38]. We detected that the N-terminal half of the protein, including NTD, makes diverse contacts throughout the entire protein (S7A Fig). There was also an abundance of contacts between the regions immediately flanking the CTD on either side, referred to as R1 (amino acids 235 to 256) and R2 (amino acids 369 to 390).

**Fig 3:**
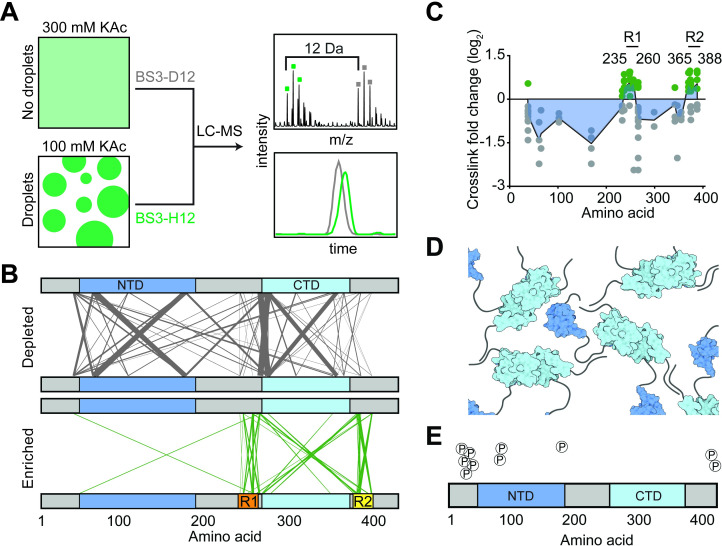
CLMS reveals interdomain interactions of the N protein. **(A)** Schematic of the CLMS experiment. (Left) A high salt (300 mM KAc) buffer disrupts N condensates, whereas a low salt (100 mM KAc) buffer promotes condensate formation. (Right, top) Example of an individual cross-linked peptide in quantitative CLMS analysis. Precursor ions from the high salt (gray) and low salt (green) BS3 cross-linking conditions show the 12 Da shift between light (H12) and heavy (D12) cross-linkers. (Right, bottom) Ion chromatograms from the first 3 isotopes of each doublet were extracted and expressed as the ratio of peak areas. **(B)** The plot of cross-links depleted and enriched in the condensate condition. The width and transparency of the lines scale with the number of times the cross-link were detected across 3 independent experiments. **(C)** Fold changes of cross-link abundance upon condensate formation of N. As cross-links contain 2 positions, fold change information is plotted at both positions. Only cross-links with *p*-values less than 0.05 are included. Green and gray dots represent cross-links enriched and depleted in the condensate condition, respectively. The blue area represents a plot of median cross-link fold change. Data underlying this figure can be found in S1 Table. **(D)** Model for how multiple N dimers could phase separate via their disordered regions. **(E)** Phosphorylation sites detected by the CLMS experiment in 300 mM KAc. CLMS, cross-linking mass spectrometry; CTD, carboxyl-terminal domain; N, nucleocapsid; NTD, N-terminal domain.

Next, we performed quantitative CLMS measurements [60] comparing the soluble N protein in 300 mM KAc with phase-separated N protein in 100 mM KAc (Fig 3A). The soluble N protein was cross-linked with heavy (D12) BS3, whereas the phase-separated protein was cross-linked with light (H12) BS3. As a result, the cross-linked precursor ions from high and low salt conditions were spaced by 12 Da (Fig 3A). Interactions between specific regions that promote condensate formation are implicated by the ratio of the cross-linked precursor ion signal and its corresponding isotopic doublet (Fig 3A) [60]. This experiment was repeated by reversing the labels, such that (H12) BS3 was used to cross-link the soluble N protein and (D12) BS3 was used for the phase-separated N protein. Across 2 independent experiments, 29 unique cross-links were enriched, and 30 cross-links were depleted upon phase separation (Fig 3B–3C, S7B Fig). Although lysine residues are distributed throughout the N protein (S7A Fig) and depleted cross-links spanned the entire primary sequence, the analysis of the cross-link fold change (S1 Table) revealed that nearly all of the enriched interactions are concentrated in regions R1 and R2 (Fig 3C). These results suggest that the interactions involving the amino acids in regions R1 and R2 may be important in driving phase separation (Fig 3D, S7C Fig).

Our mass spectrometry (MS) analysis also found phosphorylation sites on the N protein (Fig 3E). While some of these sites have been identified in previous studies [10,61,62], we also identified several novel sites (S2 Table). Although one of the phosphorylation sites (S176) is involved in a cross-link, the phosphorylated and unphosphorylated peptides were both strongly depleted in condensates, suggesting that S176 phosphorylation does not play a major role in phase separation (S1 and S2 Tables, S7B Fig). Additionally, MS identified native proteins that co-purified with the N protein in 1 M salt (S3 Table). Consistent with the recruitment of N to stress granules in cells [1,13,46, 63], 2 of the most frequently identified proteins were stress granule proteins G3BP1 and G3BP2, with R2 of the N protein interacting with G3BP1 (S7D Fig).

### The carboxyl-terminal region and phosphorylation modulate phase separation

**Fig 4:**
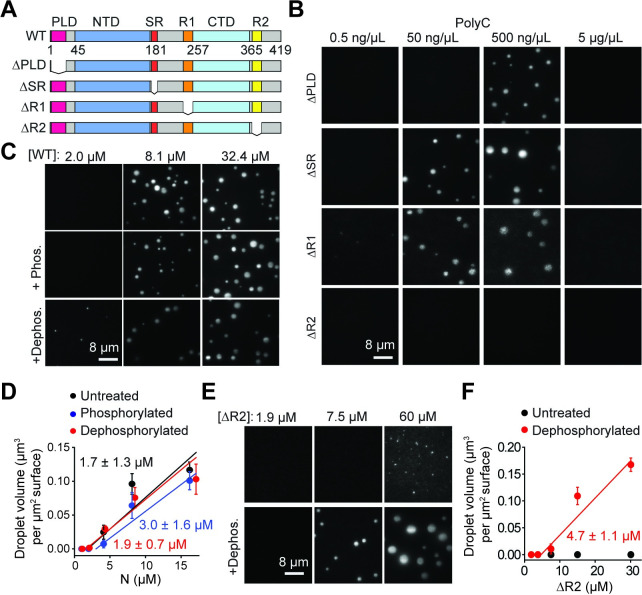
The effect of domain deletions and phosphorylation on phase separation of the N protein. **(A)** Deletion mutants of the N protein. **(B)** While ΔPLD, ΔSR, and ΔR1 phase separate, ΔR2 does not phase separate when mixed with polyC RNA. Protein concentration was set at 18 μM for all conditions. **(C)** Images of condensates formed by untreated, phosphorylated, and dephosphorylated N protein in 50 ng/μL polyC RNA. **(D)** The total volume of N-RNA condensates settled per micron squared area on the coverslip under different phosphorylation conditions (mean ± SD, *n* = 20 with 2 technical replicates). Linear fits (solid lines) reveal c_sat_ (± SE). **(E)** Images of condensates formed by untreated and dephosphorylated ΔR2 in 50 ng/μL polyC RNA. **(F)** The total volume of ΔR2-RNA condensates settled per micron squared area on the coverslip as a function of ΔR2 concentration (mean ± SD, *n* = 20 with 2 technical replicates). The linear fit (solid line) reveals c_sat_ (± SE). Data underlying this figure can be found in S1 Data. CTD, carboxyl-terminal domain; N, nucleocapsid; NTD, N-terminal domain; PLD, prion-like domain; SR, serine/arginine-rich; WT, wild-type.

The quantitative CLMS experiments show that interactions between R1 and R2 are correlated with the formation of condensates. However, we could not exclude the possibility that some of the changes in pairwise interactions are due to differences in protein structure or electrostatic interactions induced by differences in salt concentrations used for the soluble and condensate phase. To directly test the predictions of the CLMS results, we determined how different domain deletions affected phase separation of the N protein with polyC RNA under the same salt concentration (Fig 4A, S8A Fig). The ΔSR, ΔPLD, and ΔR1 mutants formed spherical condensates with polyC RNA (Fig 4B, S9 Fig), suggesting that these regions are not essential for phase separation. In comparison, deletion of the R2 sequence fully abolished the formation of condensates with polyC RNA (Fig 4B, S9A–S9D Fig). Similarly, ΔR2 was unable to form condensates with viral RNA, whereas other deletion mutants phase separated with the same RNA fragments (S9E Fig). ΔR2 maintained a high affinity to bind polyC and viral RNA (S8C Fig), excluding the possibility that the disruption of phase separation is due to the lack of protein–RNA interactions. These results show that protein–protein interactions driven by the R2 motif are required for phase separation of N protein with RNA, consistent with the proposed role of this sequence in the oligomerization of N protein [26].

Recent studies proposed that the N protein is phosphorylated early in infection, and this results in localization of N protein with the RTC, where it enhances transcription of subgenomic RNA [64]. However, nucleocapsid formation does not require phosphorylation and N protein in SARS-CoV viruses is hypophosphorylated [64,65]. The underlying molecular mechanism and kinases and phosphatases responsible for posttranslational modification of N protein remain poorly understood [8]. To understand how phosphorylation affects phase separation, we treated the full-length N protein with casein kinase 2 and λ protein phosphatase (see Methods). While kinase treatment did not alter the migration of N protein on a denaturing gel, phosphatase treatment resulted in a reduction in molecular weight (S8A Fig), suggesting that the N protein expressed in human cells is phosphorylated. Phosphatase treatment resulted in phase separation at slightly lower concentrations in comparison to kinase-treated N protein (Fig 4C and 4D). Similar to WT N, dephosphorylated ΔSR or ΔR1 had only a moderate increase in phase separation (S8 and S10 Figs). However, unlike untreated ΔR2, dephosphorylated ΔR2 exhibited robust phase separation with polyC RNA (Fig 4E and 4F). These results suggest that phosphorylated N is capable of phase separation with RNA, primarily due to interactions between R2 motifs. In the absence of the R2 motif, phosphorylation negatively regulates phase separation of the N protein.

### Targeting phase separation of the N protein with small molecules

**Fig 5:**
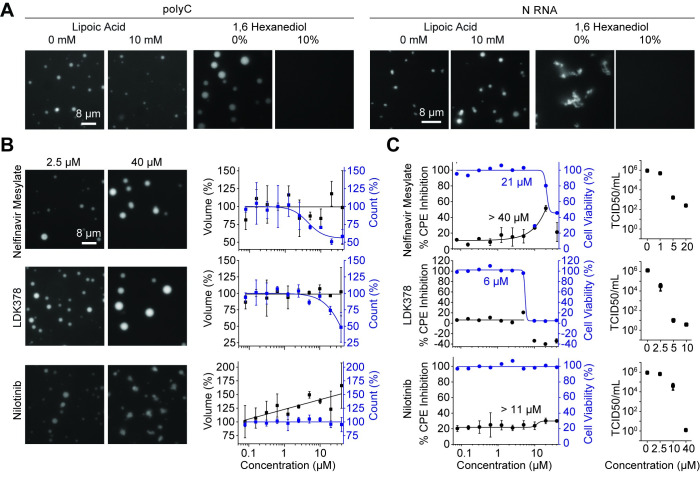
Identification of small molecules that alter phase separation of N in vitro and reduce viral titer in SARS-CoV-2 infected cells. **(A)** Condensates formed in the presence of 7.8 μM N and either 50 ng/μL polyC or 18 nM 0–5 kb viral RNA were not affected by 10 mM lipoic acid but dissolved in the presence of 10% 1,6 hexanediol. **(B)** (Left) Examples of drugs that affected N phase separation with polyC in vitro. The N protein and polyC RNA concentrations were set to 7.8 μM and 50 ng/μL, respectively. (Right) The percent change on the number (blue) and total volume (black) of N-polyC condensates settled per micron squared area on the coverslip under different drug concentrations (mean ± SD, *n* = 8 with 2 technical replicates). Solid curves represent a fit to a dose–response equation (see Methods). **(C)** (Left) Percent CPE inhibition (black, mean ± SD, 2 technical replicates) and cell viability (blue) of SARS-CoV-2–infected Vero-E6 cells treated with serial dilutions of drugs. Solid curves represent a fit to a dose–response equation (see Methods) to determine a half-maximal response constant EC_50_ (S4 Table). (Right) Viral titer in SARS-CoV-2–infected Vero-E6 cells treated with serial dilutions of drugs as measured by a TCID_50_ assay (mean ± SD, 3 technical replicates). Data underlying this figure can be found in S1 Data. CPE, cytopathic effect; N, nucleocapsid; SARS-CoV-2, Severe Acute Respiratory Syndrome Coronavirus 2; TCID, tissue culture infectious dose.

We next sought to identify small molecules that could interfere with the phase separation of the N protein. The condensates formed by the N protein in the absence or presence of polyC or viral RNA dissolve with the addition of 10% 1,6 hexanediol, indicating that phase separation is driven, at least partially, by hydrophobic interactions (Fig 5A) [45]. In comparison, lipoic acid that increases the liquidity of stress granules [66] did not affect phase separation (Fig 5A). Using a high-throughput microscopy platform, we also tested whether any of the 1,200 compounds in a Food and Drug Administration (FDA)-approved drug library alters phase separation of the N protein with polyC RNA in vitro. While none of the compounds fully dissolved the condensates, we identified several compounds that affected their number, size, and shape (S4 Table). Nelfinavir mesylate and LDK378 produced larger but fewer condensates, whereas crystal violet, tolcapone, and chlorhexidine enhanced phase separation by increasing the number and size of the condensates (Fig 5B, S11A and S11B Fig). Nilotinib resulted in a 50% increase in condensate volume and altered the shape of the condensates (Fig 5B), which may be due to changes in condensate fusion or maturation. While most drugs did not have a substantial effect on condensates formed by N and viral genomic RNA in vitro, nilotinib addition resulted in the formation of thread-like filaments (S11C and S11D Fig). Morphologically, these filaments were similar to those formed during the liquid-to-solid transition of FUS condensates [67], suggesting that nilotinib increases the viscosity of the condensates.

We then tested these drugs in human pulmonary epithelial (Calu-3) and African Green Monkey kidney (Vero-E6) cells infected with SARS-CoV-2 (see Methods), as these cell lines supported high levels of infection [68]. The cells were treated with different concentrations of drugs 1 hour before infection, and the inhibition of SARS-CoV-2–mediated cell death under drug treatment was quantified using the cytopathic effect (CPE) inhibition assay [68]. The toxicity of the drugs was quantified by measuring the viability of uninfected cells under drug treatment. Among the molecules we identified, nelfinavir mesylate resulted in the highest percent CPE inhibition in both cell lines without significantly affecting cell viability (Fig 5C, S12A Fig), with the exception that the highest dose (40 μM) was toxic to the cells.

We also tested the effectiveness of these drugs using a median tissue culture infectious dose (TCID_50_) assay. The supernatant from Vero-E6 cells that had been cultured with virus and drugs was added to uninfected cells, and the viral titer was measured by observing CPE in these cells. In this assay, nelfinavir mesylate, nilotinib, and LDK378 each reduced SARS-CoV-2 titer viability, with nilotinib and LDK378 producing an inhibitory effect similar to remdesivir, which strongly inhibits proliferation of SARS-CoV-2 in infected cells (Fig 5C, S12B Fig) [69]. Collectively, our drug screen identified compounds that affect the condensate formation of N protein and RNA in vitro, inhibit virus-mediated cell death, and reduce viral titer.

### The N protein phase separates in mammalian cells

**Fig 6:**
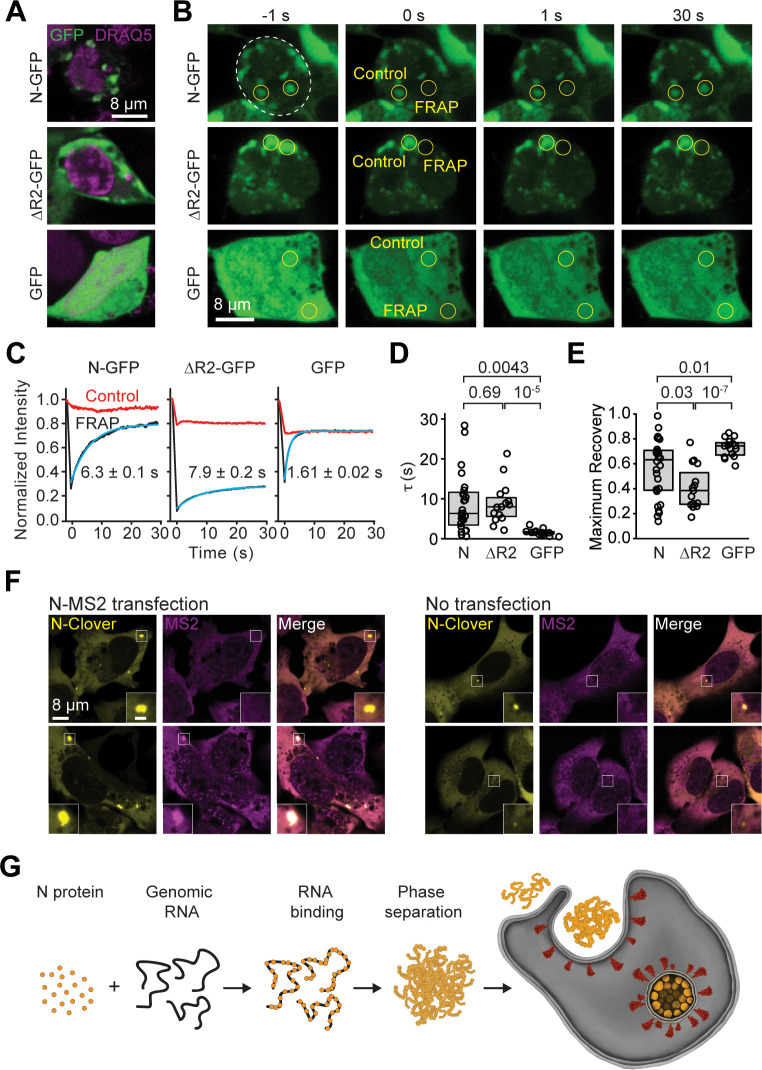
Dynamics of N condensates in vivo. **(A)** Example images of HEK293T cells expressing N-GFP, ΔR2-GFP, and GFP stained with DRAQ5. **(B)** Representative FRAP imaging of cells exhibiting N-GFP or ΔR2-GFP puncta or high GFP expression. Circles show the photobleached area. **(C)** Fluorescence recovery of the GFP signal in the bleached versus the control regions. The solid curve represents a single exponential fit to reveal the recovery lifetime (τ, ±95% confidence interval). **(D)** The distribution of fluorescence recovery lifetimes of cells expressing N-GFP (*n* = 28) or ΔR2-GFP (*n* = 15) exhibiting puncta and GFP only (*n* = 15). The center and edges of the box represent the median with the first and third quartiles. The *p*-values were calculated from a 2-tailed *t* test. **(E)** The maximum fractional recovery after photobleaching of cells expressing N-GFP (*n* = 28), ΔR2-GFP (*n* = 15), or GFP only (*n* = 15). The center and edges of the box represent the median with the first and third quartiles. The *p*-values were calculated from a 2-tailed *t* test. **(F)** U2OS cells stably expressing N-Clover form condensates in the cytoplasm. (Left) Cells co-transfected with an N-MS2 expression plasmid exhibit colocalization of N-Clover and Cy3-MS2 FISH signal in N-condensates (*N* = 10 out of 55 cells, 2 technical replicates). (Right) The Cy3-MS2 FISH probe does not partition into N condensates in untransfected cells (*N* = 55 cells, 2 technical replicates). Inset scale bar is 2 μm. **(G)** Model for remodeling of viral RNA genome by the SARS-CoV-2 N protein. The N protein packages viral genomic RNA through phase separation, which may facilitate efficient replication of the genomic RNA and the formation of the enveloped virus. Data underlying this figure can be found in S1 Data. FRAP, fluorescence recovery after photobleaching; N, nucleocapsid; SARS-CoV-2, Severe Acute Respiratory Syndrome Coronavirus 2.

To test whether N protein also phase separates in mammalian cells, we expressed N-GFP in HEK293T cells. We observed the formation of distinct puncta in the cytoplasm of N-GFP–expressing cells, which were not observed in control cells expressing GFP only (Fig 6A). FRAP imaging of these puncta revealed a recovery signal with 60% mobile fraction and 6.3 ± 0.1 seconds recovery lifetime, suggesting that N protein is capable of forming liquid condensates in cells (Fig 6B, S13A–S13D Fig). The recovery of N-GFP was substantially slower than GFP only (Fig 6C–6E), but an order of magnitude faster than that of N-polyC condensates in vitro (Fig 1H) [8,38], suggesting that these condensates are less viscous than N-polyC condensates formed in vitro. This difference is not due to the absence or low stoichiometry of RNA in N-GFP puncta in live cells because N condensates formed in the absence of RNA in vitro also exhibited an order of magnitude slower recovery than N-GFP puncta in live cells (S14 Fig). We next tested whether deletion of the R2 region disrupts phase separation of N protein in cells. Surprisingly, cells expressing the R2 deletion mutant (ΔR2-GFP) still formed puncta and did not display significantly different FRAP recovery times than N-GFP in live cells (Fig 6A–6E). This may be due to macromolecular crowding of the cellular environment because the addition of 10% polyethylene glycol (PEG) triggered phase separation of ΔR2 in vitro (S15 Fig).

Additionally, we sought to determine whether the N RNA transcript was recruited to the N condensate. We transfected U2OS cells stably expressing N-Clover [38] with a construct producing N-MS2 RNA (see Methods) and performed fluorescence in situ hybridization (FISH) assays against the MS2 RNA sequence. In the absence of N-MS2 transfection, the MS2 probe did not localize to the N-Clover puncta in any of the cells (*N* = 55 cells, 2 independent experiments) (Fig 6F). In comparison, the MS2 probe was strongly localized to all of the N-Clover condensates in approximately 18% of the cells transfected with N-MS2 (*N* = 10 in 55 cells, 2 independent experiments, Fig 6F). In other cells, we observed that the MS2 probe was uniformly distributed inside and outside of the N puncta, suggesting that these cells were not expressing N-MS2 at high enough levels to recruit the probe. We also performed RNA FISH using a d(T)20 probe that binds the polyA tails of RNA transcripts. Consistent with a previous report [38], the d(T)20 probe was not recruited to N-Clover condensates (S13E Fig). These results suggest that N-Clover condensates selectively recruit viral RNA. Differences between in vitro and in vivo phase separation properties of N protein might be attributed to macromolecular crowding of the cytosol or the interaction of the N protein with other cellular proteins, such as G3BP1 (S7D Fig) [1,46,63,70].

## Discussion

In this study, we showed that the SARS-CoV-2 N protein phase separates with viral and nonviral RNA sequences in vitro and the viscosity and shape of these condensates depend on the structure of the RNA substrate. Concurrent studies have shown that the SARS-CoV-2 N protein expressed in bacteria forms biomolecular condensates with RNA in vitro [8,31,36,38,46,54,56,63,71–74]. We purified N protein from mammalian cells to recapitulate the posttranslational modifications that occur in infected human cells [10]. Consistent with previous reports, we observed that the N protein forms liquid condensates with various RNA substrates in physiological salt [8,36]. Condensates of N with homopolymeric RNA that do not form Watson–Crick or Hoogsteen base pairing interactions recovered from photobleaching and relaxed to a spherical shape, suggesting that they are dynamic, liquid-like compartments. In agreement with Iserman and colleagues, we found that RNA structure affects the material properties of the condensate [56]; RNA capable of Watson–Crick or Hoogsteen base pairing produces irregularly shaped condensates [54,73]. N protein also formed solid-like condensates with long fragments of SARS-CoV-2 genomic RNA. These condensates had asymmetric shapes and did not relax over time or with increased temperature [8,46]. Viral RNA may drive the formation of abnormal condensate shapes by forming networks of intermolecular interactions, as previously reported [19,52,53,75].

We also investigated the mechanisms underlying condensate formation using CLMS and protein engineering [38]. Previous studies reported that prion-like and SR motifs are common features of phase separating proteins [29,33], and the linker sequence outside the SR motif (amino acids 210 to 247) is essential for phase separation of N expressed in bacteria [38]. The SR motif was required for N/N interactions in SARS-CoV and for forming puncta in SARS-CoV–infected cells [76]. In addition, the SR motif is highly phosphorylated in human cells, and phosphorylation has been shown to promote interactions with host proteins, nuclear targeting, and transcription of the viral genome and either enhances or inhibits oligomerization in SARS-CoV [13,64,65,77]. In SARS-CoV-2, SR phosphorylation is reported to make N condensates more liquid [8,38], and the deletion of the SR domain enhanced N phase separation in cells [38,63]. However, we observed that the SR region is not necessary for in vitro condensate formation of N protein expressed in human cells. The deletion of the entire carboxyl-terminal IDR was reported to enhance phase separation [8,26]. We found that deletion of the R2 motif in this region is sufficient to disrupt phase separation of N protein with both homopolymeric and viral RNA in vitro, but dephosphorylation of this mutant recovered phase separation. The carboxyl-terminal region interacts with the M protein, which was shown to drive phase separation of the N protein in the absence of RNA [26]. Because this region plays an important role in phase separation, binding to the membrane-associated M protein through this region can alter the material properties of the N-RNA condensates and initiate virion assembly.

It remains to be demonstrated whether condensate formation of N protein and the viral genome is essential for the propagation of SARS-CoV-2 in human cells. For example, several viruses have been shown to replicate in viral inclusion bodies that are characterized as phase-separated condensates [23,24]. A recent in vitro work has shown that nucleoproteins and phosphoproteins of the measles virus form liquid-like membraneless organelles and triggered nucleocapsid formation [18], suggesting that phase separation could be a general mechanism for viral replication. In the case of SARS-CoV-2, phase separation of N protein can form membrane-less compartments and function as a selectivity barrier to control the entry of certain agents into these compartments. Recent in vitro studies have shown that N-RNA condensates recruit the components of the SARS-CoV-2 replication machinery [46]. This mechanism may increase the efficiency of replication of the viral genomic RNA by increasing the local concentration of the replication machinery at the RTC complex.

N condensates may also sequester the viral assemblies from the immune response of the host cell [8]. For example, N protein interacts with stress granule proteins G3BP1 and G3BP2 in vitro [1,54,63], localizes to stress granules in cells [1,13,46,63], and inhibits stress granule formation in cells overexpressing N protein [70, 78] and in SARS-CoV-2–infected cells [79]. N mutants that do not interact with G3BP1 failed to suppress stress granule formation in SARS-CoV-2–infected cells, and these cells were impaired in mediating SARS-CoV-2 viral-like particle (VLP) production [80]. LLPS of SARS-CoV-2 N protein has also been shown to inhibit poly-ubiquitination and aggregation of mitochondrial antiviral-signaling protein (MAVS, also known as IFN-β promoter stimulator I) and thereby suppress the antiviral immune response [79]. Lys375 mutation or a peptide targeting the CTD of N protein disrupted LLPS of N with RNA in vitro, inhibited SARS-CoV-2 replication, and rescued antiviral immunity in mice [79]. Future work will be required to dissect the precise role of N-mediated phase separation in suppression of the innate immune response in infected organisms.

The phase separation of N protein with viral RNA may also facilitate the compaction and packaging of the genome into the nascent viral particle (Fig 6G) [81,82]. However, this model raises several questions. Because N protein can phase separate with nonspecific RNA substrates, it remains unclear how these condensates may exclude subgenomic viral RNA and other RNA from the host cell. Recent in vitro work has shown that N protein has a higher affinity to bind 5′ and 3′ untranslated regions (UTRs) of the genomic RNA [56,71], which may serve as a mechanism to trigger genome packaging and exclude other RNA from the condensates. In addition, condensates observed in vitro and in vivo are large structures that can potentially contain thousands of N proteins and RNAs, and it is not clear how a virion with a single genomic RNA can bud from these structures. A recent modeling study proposed that the presence of high-affinity sites in genomic RNA can trigger the formation of single-genome condensates [31]. Alternatively, interaction with the M protein or dephosphorylation of N protein, which could trigger the liquid-to-solid transition of N-RNA condensates [8,38], at the viral assembly sites may trigger budding of a single genomic copy in virions. A condensate formed by N and a single genomic copy is expected to be stabilized once encapsulated in a lipid bilayer of the virion, and this would allow the virus to maintain its genome organization in a dilute phase. Testing of these models requires in-depth studies of N protein and genomic RNA in SARS-CoV-2–infected cells.

Phase separation could also provide a macroscopic readout to study N protein and RNA interactions [66] and suggests novel strategies to disrupt genome packaging and viral propagation in infected cells. We performed a screen of an FDA-approved drug library and identified several compounds that altered the size, number, and shape of N/RNA condensates in vitro. In particular, nelfinavir mesylate binds the SARS-CoV-2 protease [83–86], and both nelfinavir mesylate and nilotinib were shown to block SARS-CoV viral production [87,88]. We showed that nelfinavir mesylate, nilotinib, and LDK378 each inhibit the proliferation of SARS-CoV-2 in the host cell, which may be related to changes they induce on N condensates. Future work in infected cells is needed to address whether these drugs reduce virus-mediated cell death by interfering with the functions of N-RNA condensates.

## Methods

### Protein purification

A construct for expressing N protein with a carboxyl-terminal Strep-tag was obtained from the Krogan lab (UCSF). A carboxyl-terminal yBBr labeling site was added for labeling with a fluorescent dye. Deletion mutants, ΔPLD (amino acids 2 to 30: SDNGPQNQRNAPRITFGGPSDSTGSNQNG), ΔSR (amino acids 183 to 195, SSRSSSRSRNSSR), ΔR1 (amino acids 235 to 256, SGKGQQQQGQTVTKKSAAEASK), and ΔR2 (amino acids 369 to 390, KKDKKKKADETQALPQRQKKQQ) were generated using Gibson cloning (New England Biosciences, Massachusetts, USA). Sufficient amounts of DNA were obtained by growing 1 L of transfected XL1 Blue *Escherichia coli* cells overnight and performing a gigaprep (Zymo, California, USA). For protein expression, HEK293S GNTI- cells (RRID: CVCL_A785) were grown in suspension in Freestyle media (Thermo Fisher Scientific, Massachusetts, USA) supplemented with 2% fetal bovine serum (FBS, VWR, Pennsylvania, USA) and 1% penicillin-streptomycin (PS, Gemini Bio Products, California, USA) to 2 million cells/mL. Cells were spun down for 10 minutes at 1,200 *g* and resuspended in fresh, antibiotic-free media. For 250-mL cells, the transfection solution was created by mixing 1.8-mL polyethylenimine (1 mg/mL, pH 7.0 in PBS) dissolved in 20-mL Freestyle media and 0.66-mg DNA dissolved in 20-mL Freestyle media. The mixture was incubated at room temperature for 15 minutes before being adding it to the cell culture. Transfected cell culture was grown for 72 hours at 125 rpm at 37°C with 5% CO_2_ and 5% humidity.

Cells were then harvested at 4,000 *g* for 10 minutes and resuspended in 50 mL lysis buffer (50 mM HEPES pH 7.4, 1 M NaCl, 1 mM PMSF, 1 mM DTT, and 1 tablet of protease inhibitor (Sigma-Aldrich, Missouri, USA)). Lysis was performed using 15 loose and 15 tight plunges of a Wheaton glass dounce. The lysate was clarified using a 45 minutes, 360,000 *g* spin in a Ti70 rotor. The supernatant was incubated with 1-mL Streptactin sepharose beads (IBA Life Sciences, Goettingen, Germany) for 1 hour. Beads were washed with 40 mL of lysis buffer followed by 30 mL labeling buffer (50 mM HEPES pH 7.4, 300 mM NaCl, 10 mM MgCl_2_, 1 mM EGTA, 10% glycerol, 1 mM DTT). Beads were then collected and incubated with purified Sfp phosphopantetheinyl transferase and an LD655 dye functionalized with CoA (Lumidyne, New York, USA) at room temperature for 30 minutes. Beads were washed with 30-mL labeling buffer. If proteins were to be kinase or phosphatase treated, they were additionally washed with 30 mL kinase (20 mM HEPES pH 7.5, 300mM NaCl, 10 mM MgCl2, 200 μM ATP, 10% glycerol, 1 mM DTT) or phosphatase (20 mM HEPES pH 7.5, 300mM NaCl, 1mM MnSO4, 10% glycerol, 1 mM DTT) buffer. Protein was eluted in 1 mL fractions in its final buffer supplemented with 10 mM desthiobiotin and concentrated using Amicon Ultra 30K concentrators. For kinase and phosphatase treatment, 5 μL of caseine kinase 2 (New England Biolabs, Massachusetts, USA) or 2.5 μL λ phosphatase (New England Biolabs, Massachusetts, USA) was added per 50 μL concentrated protein, respectively, and the samples were incubated at 30°C for 1 hour. Final protein concentration was measured using Bradford reagent, and aliquots were snap frozen in liquid nitrogen.

### In vitro transcription and RNA labeling

For in vitro transcription of long viral RNA, the region of interest was first PCR amplified from a plasmid (N plasmid was a generous gift from the Krogan lab [1], 5′ UTR plasmid was a generous gift from the Gladfelter lab [56], and SARS-CoV-2 cDNA plasmid was a generous gift of the Thiel lab [55]) using a forward primer with a T7 polymerase binding site (TAATACGACTCACTATAGGG). The amplified DNA was tested for purity on a 0.8% agarose gel and cleaned up using GlycoBlue and ethanol precipitation. RNA was generated using the HiScribe T7 Quick kit (New England Biolabs, Massachusetts, USA) and extracted using trizol and isopropanol precipitation. RNA was Cy3 labeled using a Label IT kit (Mirus Bio, Wisconsin, USA), and RNA purity was verified using a 0.8% agarose gel. All RNA structure predictions were done on the RNAfold server [57,58].

### Sample preparation and microscopy

Purified protein and RNA samples were diluted into the imaging buffer (50 mM HEPES pH 7.4, 150 mM NaCl, 5 mM MgCl_2_, 1 mM EGTA, 1 mM DTT, 1% pluronic) to their final concentration and introduced into the flow chamber. Samples were settled onto the coverslip for 25 minutes before imaging. We confirmed that all of the condensates were settled to the surface within 25 minutes, as we did not observe an increase in the number of condensates per viewing area of the coverslip, and we could not detect freely diffusing condensates in the flow chamber after 25 minutes. Different buffers were used for phosphatase-treated (50 mM HEPES pH 7.4, 150 mM NaCl, 0.5 mM MnSO4, 1 mM DTT, 1% pluronic) and kinase-treated (20 mM HEPES pH 7.5, 150 mM NaCl, 5 mM MgCl_2_, 100 μM ATP, 1 mM DTT, 1% pluronic) samples.

Imaging was performed using a custom-built fiber-coupled Nikon Ti-E Eclipse microscope equipped with an objective-type total internal reflection fluorescence (TIRF) illuminator and 100X 1.49 N.A. Plan Apo oil immersion objective (Nikon, New York, USA). The samples were excited in near-TIRF using 561 and 633 nm laser beams (Coherent, California, USA). The fluorescent signal was detected by Andor Ixon electron-multiplying CCD (EMCCD) camera (512 × 512 pixels). The effective pixel size was 160 nm after magnification. 10 single-frame images were acquired for each condition in each replicate.

### Image analysis

To calculate the saturation concentration (c_sat_), the area of each condensate was quantified with Fiji using the Phansalkar function with a 30-pixel radius and a minimum condensate size of 10 pixels. The volume of the condensates was estimated from 2D projections by taking the semi-principal axis in the z-plane as the geometric average of semi-principal axes in the xy plane. The total volume of the condensates settled per micron squared on the coverslip was quantified. Conditions that resulted in measurable condensate volumes were fit to linear regression in Origin. The x-intercept of the linear regression represents c_sat_, the minimum protein concentration that results in condensate formation. The aspect ratio was calculated for individual condensates in Fiji using the Phansalkar function with a 30-pixel radius and a minimum condensate size of 10 pixels. To measure fusion times, condensates were visualized as they settled on the imaging surface from 5 to 25 minutes after mixing. Movies were recorded at 5 frames per second. Fusion times were calculated as the time between the last frame where 2 condensates appear separated (i.e., no overlap) and the first frame where the fused condensate appears nearly spherical (aspect ratio = 1.1).

### In vitro FRAP

In vitro FRAP assays were performed with a Zeiss 880 Confocal Laser Scanning Microscope equipped with a 100×, 1.4 NA oil immersion objective. Samples were prepared as described above. All in vitro FRAP experiments used the 488 nm laser at 100% for bleaching conditions. For N-polyC at 150 mM NaCl, 5 bleaching iterations were used, and images were taken every 2 seconds for 232 seconds. For viral RNA at 150 mM NaCl, 100 bleaching iterations were used, and images were taken every 10 seconds for 8 minutes. For the experiments conducted at 50 mM NaCl, droplets containing polyC RNA or viral RNA were bleached with 20 iterations, and images were taken every 5 seconds for 5 minutes. For droplets containing N protein only, 20 bleaching iterations were used, and images were collected every 2 seconds for 6.5 minutes. All data were acquired using Zeiss Zen 2.3 SP1 FP3 (black) (64bit) Version 14.0.20.201. Data were background corrected, converted to normalized intensity, and fit to an exponential decay function in Origin.

### MS

The cross-linking analysis was performed as described by McGilvray and colleagues [90] with deviations outlined here. 15 μL 90 μM N protein in the labeling buffer was diluted 1:3 v/v into either water or labeling buffer. The diluted protein formed condensates in 100 mM NaCl in the final buffer, but not in the labeling buffer that contains 300 mM NaCl. Isotopically coded light (H12) and heavy (D12) BS3 (bis(sulfosuccinimidyl)suberate) cross-linkers (Creative Molecules, California, USA) were immediately added to the diluted solution. Final concentration of BS3 was 0.8 mM for 100 mM NaCl dilution and 2.5 mM for 300 mM NaCl dilution. Cross-linking was performed for 30 minutes at room temperature and then quenched with 1 M Tris pH 8.0 buffer. This experiment was performed in duplicate, once with D12-BS3 cross-linking N protein in condensates and H12-BS3 cross-linking soluble N protein and once with the labels reversed. The cross-linked proteins from both channels were pooled before acetone precipitation. Protein was precipitated with acetone overnight at −20°C. Protein was pelleted, the supernatant was removed, and the samples were air-dried for 10 minutes. The pellets were brought up in 8 M urea, reduced with TCEP, alkylated with iodoacetamide, diluted 4-fold, and digested with 2 rounds of trypsin. Peptides were desalted on a C18 MacroTrap column (Michrom Bioresources, California, USA) and fractionated on a Superdex Peptide (GE Life Sciences, Illinois, USA) size exclusion column. Fractions enriched in cross-linked peptides were dried and resuspended in 0.1% formic acid for MS. Later eluting fractions were used for phosphorylation analysis. Each of the replicates was fractionated by size exclusion chromatography (SEC), and each fraction was injected twice, generating 16 MS files for analysis.

MS was acquired on an Orbitrap Fusion Lumos coupled with an Easy-Spray nanoelectrospray ion source, a 15 cm × 75 μm PepMap C_18_ column (Thermo Fisher Scientific, Massachusetts, USA) and an M-class NanoAcuity UPLC system (Waters, Massachusetts, USA). Liquid chromatography–mass spectrometry (LC–MS) runs were 90 minutes long. Precursor ions were measured in the Orbitrap at 120 k resolution. Selected precursor ions (triply charged and higher) were isolated, and the product ions were measured in the Orbitrap at 30 k resolution. Samples that were analyzed for phosphorylation were run similarly except only HCD product ions spectra were collected and doubly charge precursors were included.

Cross-linked spectra were identified with Protein Prospector 6.2.23 [91] using the combination of DSS/DSS:2H12 at uncleaved Lys residues and protein N-terminus as the cross-linking reagents. The corresponding light and heavy dead-end modifications, incorrect monoisotopic peak assignment (+1Da neutral loss), N-terminal pyroglutamate formation, methionine oxidation, and acetylation and loss of the protein N-terminal methionine were set as variable modifications with 3 variable modifications per peptide allowed. Trypsin with 2 missed cleavages was the digestion enzyme, and the mass tolerances were 20 and 30 ppm for precursor and product ions. The search database comprised the 14 most abundant proteins (sorted by spectral abundance factor, SAF) found in the linear peptide SEC fractions, alongside a decoy database that was 10 times longer. Cross-link spectral matches were classified at a 1% false discovery rate (FDR) threshold.

Protein Prospector was used to search for phosphopeptides of the linear peptide fractions using the following parameters: tryptic specificity with 2 missed cleavages, 7 and 15 ppm precursor and product ion tolerances, and carbamidomethyl (C) fixed modification. Variable modifications were as above except that Phospho (STY) was included, and only dead-end cross-link modifications were included. Peptides were reported with a maximum expectation value of 0.001, and a minimum prospector score of 15. A site localization in peptide (SLIP) score threshold of 6 was used to determine site localization. All phosphopeptide spectra reported were manually inspected for evidence of correct site assignment. Where S or T residues are adjacent, the location of the phosphosite is ambiguous (see S2 Table). All phosphorylation data can be accessed here (or on the MS-Viewer website with search key “jyovxenjny”).

A supplemental search of the cross-linked peak lists was made for cross-links that also contained a phosphorylation site. This search was identical to the previous cross-linking searches except that Phospho (ST) was included as a variable modification, and the search was limited to the N protein sequence. Search results containing a phosphorylation site were manually assessed. Only phosphorylation sites that had been discovered in the phospho search of the linear peptides were considered.

For quantitating the isotopically labeled cross-links, peak areas were measured from the extracted precursor ion chromatograms (XICs) using the small molecule interface of Skyline (v20.1.0.155). A Skyline transition list was generated containing the elemental composition of each distinct peptide pair with both light and heavy BS3 modification and in each charge state detected in the Prospector search. Retention times were present in the transition list to help with peak detection. Peptide level measurements were imported into R and summarized at the level of unique residue pairs (“cross-links”). For each precursor ion, the log_2_ ratios of heavy to light peptides were calculated and mean normalized to 0 for each of the 2 biological replicates and then transformed into log_2_ (water/salt) ratios. Weighted *t* tests were performed in R.

### Drug screening and image processing

For the FDA-approved drug screen, 75 mL of N protein at 16 μM was purified from HEK293S GNTI- cells. polyC RNA was obtained from Sigma. The FDA-approved drug library (TargetMol) has 1,200 compounds of well-characterized biological activity. A total of 10 mM compounds were stored in 100% dimethyl sulfoxide (DMSO) in 384-well plates. For screening plates, 20 μL imaging buffer (50 mM HEPES pH 7.4, 150 mM NaCl, 5 mM MgCl_2_, 1 mM EGTA, 1 mM DTT, 1% pluronic) was aliquoted into 384-well, glass-bottomed plates (Greiner Bio-One, Kremsmunster, Austria) and incubated for 5 minutes. Buffer was removed, and 0.5 μL of 2 mM compounds were stamped into 384-well plates with an Analytik-Jena Cybio Well Vario liquid handler. The final concentration of 7.8 μM N protein and 50 ng/μL polyC RNA were added to each plate. Each well contained a 25 μL mixture and 40 μM compound in the primary screen.

Wells were homogenized with a Bioshake 3000 ELM orbital shaker at 2,400 rpm for 45 seconds, and condensates were allowed to settle for 1 hour before imaging. Moreover, 4 images (each 224 × 167 μm) were taken at 40× with ImageXpress Micro High Content Imaging System (Molecular Devices, California, USA). Images were analyzed with Metamorph Imaging software calculating condensate count and area. The volume of the condensates was calculated as described above. Data were then uploaded to CCD Vault for normalization to DMSO vehicle control wells. Wells that exhibit 3 SDs from the untreated sample and as well as others that produced qualitative morphological changes in N condensates were selected for dose–response screening. The same N protein and RNA were tested against candidate drugs using a 10-point serial dilution starting at 40 μM using the same procedure. Dose–response curves were generated by fitting to the equation


$$y(C)=y_{min}+\frac{\left(y_{max}-y_{min}\right)}{1+{\left(\frac{C}{{EC}_{50}}\right)}^{n}},$$

where *C* is the concentration of the drug, *n* is the Hill coefficient, *EC*_*50*_ is the half-maximal response concentration, and *y* is either the volume of the N condensates or the number of N condensates settled onto per micron squared area on the coverslip.

### Dose–response with SARS-CoV-2–infected cells

Vero-E6 (ATCC, CRL-1586) and Calu-3 cells (ATCC HTB-55) were cultured in high glucose DMEM (Gibco) supplemented with 10% FBS (R&D Systems), 1X GlutaMAX (Gibco), and 1X PenStrep (Gibco) at 37°C and 5% CO_2_. For screening selected drugs against infected cells, 2,500 Vero-E6 (12 μL/well) or 10,000 Calu-3 (12 μL/well) were seeded in 384-well white optical bottom tissue culture plates (Nunc) with the Multidrop Combi liquid handling instrument (Thermo Fisher Scientific). Cells were allowed to recover for 24 hours for Vero-E6 and 48 hours for Calu-3 at 37°C and 5% CO_2_. Dose responses were generated by diluting the compounds using a Cybio Well Vario liquid handler (Analytik Jena, Jena, Germany), leading to a final concentration of DMSO at 0.4% in the assay plate (v/v). Cells were incubated at 37°C and 5% CO_2_ for 1 hour before infection. The viral inoculum was prepared such that the final multiplicity of infection (MOI) was 0.05 upon addition of 6 μL/well viral inoculum. After complete CPE was observed in DMSO-treated, infected wells (72 hours postinfection (hpi) for Vero-E6 and 96 hpi for Calu-3), the plates were developed with the CellTiter-Glo 2.0 reagent (Promega, Wisconsin, USA) according to the manufacturer’s instructions. For Vero-E6, the reagent was diluted 1:1 (v/v) in PBS. Luminescence of developed plates was read on a Spectramax L (Molecular Devices). Each plate contained 24 wells uninfected/DMSO treated cells (100% CPE inhibition) and 24 wells infected/DMSO treated cells (0% CPE inhibition). Average values from those wells were used to normalize data and determine % CPE inhibition for each compound well. To determine the cytotoxicity of the compounds, the same protocol was used but with 6-μL growth media added instead of viral inoculum. The data were plotted and analyzed in Origin and fit to the dose–response function above.

### Dose–response determination by TCID_50_ assay

Vero-E6 cells were plated at 10,000 cells per well in Vero growth medium as above in 96-well plates and allowed to recover for 24 hours. Drug treatments were prepared in the same medium at 1.33× of final concentration and 0.53% v/v DMSO. Growth medium was removed from cells and replaced with 150-μL medium with drug treatment and incubated for 1 hour. Virus in 50-μL medium was added to each well to produce a final MOI of 0.05, final drug concentrations as indicated, and 0.4% v/v DMSO per well. Vehicle-only treatment was used as a positive control for productive viral infection and CPE, and remdesivir at 50 μM was used as a positive control for viral restriction by drug treatment. Each condition was plated in triplicate. Virus titer following drug treatment was determined by TCID_50_ assay in Vero-E6 cells. At 24 hours post infection, 100 μL of supernatant from drug-treated infected cells was removed from each well and used to prepare a 10-fold dilution series in Vero growth medium. Moreover, 50 μL of the dilution series was added to Vero cells in 96-well plates, prepared as above, in 100 μL medium. Cells were observed for CPE for 3 days. TCID_50_/mL results were calculated using the Spearman and Kärber method [92].

### HEK293T cell culture and transfection

HEK293T (RRID: CVCL_0063) cells were cultured in phenol-negative DMEM media supplemented with 10% FBS and 1% PS at 37°C with 5% CO_2_. Prior to imaging, cells were transferred to glass-bottomed plates (Nunc Lab-Tek, 0.4 mL working volume) at approximately 25% confluence and allowed to recover for 24 hours. Media was exchanged into phenol-negative DMEM media supplemented with 10% FBS, and cells were transfected with N-GFP, NΔR2-GFP, or GFP only constructs. For each well, 400 ng of DNA was added to 20 μL DMEM media. A total of 1.2 μL FuGENE HD transfection reagent (Promega) was added, and the mixture was incubated at room temperature for 15 minutes before being added to the cell culture. Cells were allowed to express the constructs for 4 days. One hour prior to imaging, DRAQ5 stain at 1:1,000 was added to the media.

### In vivo imaging and FRAP assays

In vivo imaging and FRAP measurements were performed with a Zeiss 880 Confocal Laser Scanning Microscope equipped with a 63×, 1.4 NA oil immersion objective. Samples were prepared as described above. All in vivo FRAP experiments used the 488 nm laser at 100% for bleaching conditions. Cells expressing N-GFP, ΔR2-GFP, or GFP only were bleached with 30 iterations, and images were collected continuously at 6.7 Hz for 30 seconds. All data were acquired using Zeiss Zen 2.3 SP1 FP3. Data were converted to the normalized intensity and fit to an exponential decay function in Origin.

### U2OS cell culture, transfection, and RNA FISH

U2OS cells stably expressing SARS-CoV-2 N protein with a carboxyl-terminal Clover tag were a gift from the Cleveland lab at the University of California San Diego (UCSD). Cells were cultured in phenol-negative DMEM media supplemented with 10% FBS and 1% PS at 37°C with 5% CO_2_. Prior to imaging, cells were transferred to glass-bottomed plates (Nunc Lab-Tek, 0.4 mL working volume) at approximately 25% confluence. Cells were transfected with a construct expressing N-MS2 RNA (the N cDNA sequence with 6 upstream stop codons and 3 carboxyl-terminal MS2 repeats) as described above. Cells were induced with doxycycline at a final concentration of 1,000 ng/mL and allowed to express for 14 hours. FISH was performed following the Stellaris RNA FISH protocol for adherent cells. Cells were fixed with 3.7% formaldehyde, permeabilized with 70% ethanol, and incubated in the dark at 37°C overnight with hybridization buffer (Biosearch Technologies, Middlesex, UK) supplemented with 10% deionized formamide and either 125 nM Cy3-(d)T20 oligonucleotides (Gene Link, Florida, USA) or 1 μM Cy3-MS2 probe (IDT (Iowa, USA), sequence AGGCAATTAGGTACCTTAGG) [19]. Cells were washed with wash buffer A (Biosearch Technologies, Middlesex, UK) for 30 minutes, then incubated with DRAQ5 1:1,000 in wash buffer A for 30 minutes and exchanged into wash buffer B (Biosearch Technologies) before imaging.

## Supporting information

S1 Fig Gel filtration and EMSA analysis of the WT N protein purified from HEK293S GNTI- cells. **(A)** The coomassie-stained denaturing gel of the N protein purified from affinity chromatography. **(B)** UV absorbance of the N protein purified from affinity chromatography shows no evidence for the presence of contaminating nucleic acids. **(C)** UV absorbance of protein standards eluting from a gel filtration column. **(D)** UV absorbance of the N protein eluting from a gel filtration column. Arrows mark the void volume and expected elution volume for an N monomer. **(E)** The coomassie-stained denaturing gel of the eluents from the gel filtration column. The yellow arrowhead shows the expected molecular weight of full-length N protein. **(F)** EMSA using no RNA, 10 nM 0–5 kb viral RNA, and 50 ng/μL polyC RNA and decreasing concentration of N protein. The protein was labeled with LD655. RNA was labeled with Cy3. Arrowheads indicate the minimum protein concentration for each condition with a noticeable signal in the protein gel. EMSA, electrophoresis mobility shift assay; N, nucleocapsid; WT, wild-type.
(TIF)

S2 Fig RNA substrates do not form condensates in the absence of N protein. **(A)** An agarose gel picture of 0–5 kb viral RNA and polyC RNA substrates. The gel was stained with GelRed (left), and the RNA substrates were labeled with Cy3 (right). The ladder corresponds to the length of double-stranded DNA. Estimated lengths of 0–5 kb viral RNA and polyC RNA are 5 kb and 2 kb, respectively. **(B)** Representative pictures show that 18 nM 0–5 kb viral RNA and 50 ng/μL polyC RNA do not form condensates in the absence of N protein. The assay was performed in 150 mM NaCl. **(C)** Two-color imaging shows colocalization of LD655-labeled N protein and Cy3-labeled polyC RNA in condensates. N, nucleocapsid.
(TIF)

S3 Fig FRAP analysis of N-polyC condensates in vitro. **(A)** Control experiments show changes in the fluorescent signal of N and polyC without photobleaching. **(B)** The changes in the integrated fluorescent intensities of regions highlighted with yellow and red rectangles in (A). **(C)** The maximum fractional recovery of N and polyC in condensates after photobleaching (*n* = 36, mean ± SD). The center and edges of the box represent the median with the first and third quartiles. The *p*-value was calculated from a 2-tailed *t* test. Data underlying this figure can be found in S1 Data. FRAP, fluorescence recovery after photobleaching; N, nucleocapsid.
(TIF)

S4 Fig The N protein forms asymmetric condensates with viral RNA. **(A)** Structure prediction of each section of SARS-CoV-2 genomic RNA. **(B)** The formation of asymmetric N condensates under different viral RNA concentrations. The N protein concentration was set to 18.5 μM. **(C)** Structure prediction of viral RNA (left) and the formation of asymmetric N condensates under different RNA concentrations (right). The N RNA is the 1.3 kb long genomic RNA fragment that encodes the SARS-CoV-2 N protein. The 5′ UTR RNA is the first 1,000 bases of the SARS-CoV-2 genome. The N protein concentration was set to 18.5 μM. **(D)** The distribution of aspect ratios of individual condensates formed with different RNA substrates. The N protein concentration was set to 18.5 μM, and RNA concentration was set to 50 ng/μL for RNA homopolymers and 18 nM for 0–5 kb viral RNA. The center and edges of the box represent the median with the first and third quartiles. *p*-Values are calculated from 2-tailed *t* tests. Data underlying this figure can be found in S1 Data. IVT, in vitro transcribed; N, nucleocapsid; SARS-CoV-2, Severe Acute Respiratory Syndrome Coronavirus 2; UTR, untranslated region.
(TIF)

S5 Fig Condensates formed by the N protein and viral RNA do not fuse or change shape but are dissolved at higher salt. **(A)** Condensates formed by the N protein with in vitro transcribed N RNA or the 5′ UTR of the viral RNA are not sensitive to an increase of temperature to 37°C. **(B)** Condensates formed by the N protein with 5′ UTR of viral RNA do not change shape over 24 hours. **(C)** Condensates formed by 11.5 μM N protein and 36 nM 5′ UTR RNA are dissolved in the presence of 260 mM NaCl. **(D)** Condensates formed by 18.5 μM N protein and 18 nM 0–5 kb viral RNA do not fuse after contact (yellow arrow). N, nucleocapsid; UTR, untranslated region.
(TIF)

S6 Fig FRAP analysis of N and 0–5 kb viral RNA condensates in vitro. **(A)** Control experiments show snapshots of LD655-labeled N and Cy3-labeled 0–5 kb viral RNA without photobleaching. **(B)** The changes in the integrated fluorescent intensities of the regions that are highlighted with yellow and red rectangles in **(A)**. Data underlying this figure can be found in S1 Data. FRAP, fluorescence recovery after photobleaching; N, nucleocapsid.
(TIF)

S7 Fig Interactions between N proteins and between N and G3BP1. **(A)** In an initial qualitative experiment, pairwise interactions were detected by CLMS at 300 mM salt. Blue dots depict the positions of lysine residues. Lines depict a unique cross-link detected. The regions of N protein interactions flank the CTD. **(B)** Volcano plot of the quantitative CLMS data comparing the condensate and no condensate condition. Opaque data points have a *p*-value below 0.05, and transparent data points have a *p*-value greater than 0.05. Green represents unique cross-links that are enriched in the condensate condition. The yellow markers represent the K169-K65 and K169 (phosS176)-K65 cross-links. **(C)** The structure of the CTD of the SARS-CoV-2 N protein was plotted with BioRender (PDB 6WJI [89]). Because the N terminus and carboxyl terminus of the protomers are positioned away from each other, R1 and R2 within the same dimer are unlikely to interact with each other. **(D)** MS identified that the RNA binding domain of the stress granule protein G3BP1 interacts with the R2 region of N. Data underlying this figure can be found in S1 Table. CLMS, cross-linking mass spectrometry; CTD, carboxyl-terminal domain; MS, mass spectrometry; N, nucleocapsid; SARS-CoV-2, Severe Acute Respiratory Syndrome Coronavirus 2.
(TIF)

S8 Fig Purification and characterization of the deletion mutants. **(A)** Denaturing gel pictures of purified WT and deletion mutants of N protein in the presence and absence of kinase and phosphatase treatment (see Methods). The gels were stained with Coomassie. Yellow arrowheads highlight a reduction in molecular weight upon treatment with λ phosphatase. **(B)** UV absorbance of the ΔR2 mutant eluting from a gel filtration column. UV absorbance of WT N under the same experimental conditions is shown in a dashed red curve for comparison. **(C)** EMSA gels using no RNA, 10 nM 0–5 kb viral RNA, or 50 ng/μL polyC RNA and decreasing concentration of the ΔR2 mutant. The protein was labeled with LD655. RNA was labeled with Cy3. Arrows indicate the minimum protein concentration for each condition with a noticeable signal in the protein gel. EMSA, electrophoresis mobility shift assay; MS, mass spectrometry; N, nucleocapsid; WT, wild-type.
(TIF)

S9 Fig The in vitro phase separation behavior of the deletion mutants of the N protein. **(A)** Example pictures show that the N deletion mutants, except ΔR2, form spherical condensates with polyC RNA under different RNA concentrations. The N protein concentration was set to 18.5 μM. **(B)** The total volume of N-RNA condensates settled per micron squared area on the coverslip (mean ± SD; *n* = 20, 2 technical replicates) exhibits a reentrant behavior under an increasing RNA concentration. **(C)** Example pictures show that the N deletion mutants, except ΔR2, form spherical condensates with polyC RNA under different protein concentrations. The polyC RNA concentration was set to 50 ng/μL. **(D)** The total volume of N-RNA condensates settled per micron squared area on the coverslip (mean ± SD; *n* = 20, 2 technical replicates) under an increasing protein concentration. **(E)** Phase separation of truncated N protein constructs with 0.74 μM in vitro transcribed N RNA or 18 nM 0–5 kb viral RNA. The protein concentration was set to 18.5 μM. Data underlying this figure can be found in S1 Data. N, nucleocapsid.
(TIF)

S10 Fig Phase separation of truncated N proteins under different phosphorylation conditions in vitro. **(A)** Images of condensates formed by untreated and dephosphorylated ΔSR in the presence of 50 ng/μL polyC RNA. **(B)** The total volume of the condensates settled per micron squared area on the coverslip as a function of ΔSR concentration (mean ± SD, *n* = 20 with 2 technical replicates). **(C)** Images of condensates formed by untreated and dephosphorylated ΔR1 in the presence of 50 ng/μL polyC RNA. **(D**) The total volume of the condensates settled per micron squared area on the coverslip as a function of ΔR1 concentration (mean ± SD, *n* = 20 with 2 technical replicates). **(E)** Full-length N protein forms asymmetric condensates with 0.74 μM in vitro transcribed N RNA before and after phosphorylation and dephosphorylation. The protein concentration was set to 35 μM. Data underlying this figure can be found in S1 Data. N, nucleocapsid.
(TIF)

S11 Fig Phase separation of the N protein under drug treatment. **(A)** Example pictures show phase separation of 7.8 μM N protein and 50 ng/μL polyC RNA at different drug concentrations. **(B)** The percent change on the number (blue) and total volume (black) of N-polyC condensates settled per micron squared area on the coverslip under different drug concentrations (mean ± SD, *n* = 8 with 2 technical replicates). **(C)** Example pictures show phase separation of 57.6 μM LD655-labeled N protein and 1.5 μM Cy3-labeled in vitro transcribed N RNA in the presence of different drug treatments. The concentration of drugs was set to 40 μM. **(D)** Condensates formed in the presence of 57.6 μM N protein and 1.5 μM N RNA form thread-like filaments at high nilotinib concentrations. Data underlying this figure can be found in S1 Data. N, nucleocapsid.
(TIF)

S12 Fig The viability of SARS-CoV-2–infected cells under drug treatment. **(A)** Percent CPE inhibition (black, mean ± SD, 2 technical replicates) and cell viability (blue) of SARS-CoV-2–infected Calu-3 cells treated with serial dilutions of drugs. Solid curves represent a fit to a dose–response equation to determine EC_50_. **(B)** Viral titer in uninfected and SARS-CoV-2–infected Vero-E6 cells as measured by a TCID_50_ assay. Infected cells were treated with DMSO (negative control) and 50 μM remdesivir (positive control). Data underlying this figure can be found in S1 Data. CPE, cytopathic effect; SARS-CoV-2, Severe Acute Respiratory Syndrome Coronavirus 2; TCID, tissue culture infectious dose.
(TIF)

S13 Fig Dynamics of N protein in cells. **(A)** Representative FRAP imaging of HEK293T cells with high expression of N-GFP or ΔR2-GFP. Circles show the photobleached and control (not bleached) regions. **(B)** Fluorescence recovery signals of the N protein in the bleached versus the control regions. The solid curve represents a single exponential fit to reveal the recovery lifetime (τ, ±95% confidence interval). **(C)** The distribution of fluorescence recovery lifetimes of cells expressing N-GFP or ΔR2-GFP and exhibiting either puncta or high expression (from left to right, *n* = 28, 34, 15, and 28). The center and edges of the box represent the median with the first and third quartiles. The *p*-values were calculated from a 2-tailed *t* test. **(D)** The maximum fractional recovery after photobleaching of cells expressing N-GFP or ΔR2-GFP and exhibiting either puncta or high expression (from left to right, *n* = 28, 34, 15, and 28). The center and edges of the box represent the median with the first and third quartiles. The *p*-values were calculated from a 2-tailed *t* test. **(E)** U2OS cells stably expressing N-Clover form condensates in the cytoplasm. Cy3-d(T)20 FISH probe targeting the polyA tails of RNA transcripts is uniformly distributed in cells exhibiting N condensates with (left) or without (right) expression of N-MS2 (*N* = 40 cells, 2 technical replicates). Inset scale bar is 2 μm. Data underlying this figure can be found in S1 Data. FRAP, fluorescence recovery after photobleaching; N, nucleocapsid.
(TIF)

S14 Fig Dynamics of N protein in 50 mM NaCl in vitro. **(A)** Representative FRAP imaging of N protein only, or in the presence of polyC or 0–5 kb viral RNA. The concentrations of N, polyC, and 0–5 kb viral RNA were kept at 24 μM, 50 ng/μL, and 18 nM, respectively. Rectangles highlight the photobleached area. **(B)** Fluorescence recovery signals of the N protein and RNA in the bleached versus the control regions. Solid curves represent a single exponential fit to reveal the recovery lifetime (τ, ±95% confidence interval). **(C)** The distribution of fluorescence recovery lifetimes of the condensates (from left to right, *n* = 20, 17, 23, 28, and 28). The center and edges of the box represent the median with the first and third quartiles. The *p*-values were calculated from a 2-tailed *t* test. **(D)** The maximum fractional recovery after photobleaching (from left to right, *n* = 20, 17, 23, 28, and 28). The center and edges of the box represent the median with the first and third quartiles. The *p*-values were calculated from a 2-tailed *t* test. Data underlying this figure can be found in S1 Data. FRAP, fluorescence recovery after photobleaching; N, nucleocapsid.
(TIF)

S15 Fig In vitro phase separation of WT N and deletion mutants in the presence of a crowding agent. **(A)** (Left) Images of the LD655-labeled N protein in the presence of 10% PEG and 150 mM NaCl. The assays were performed in the absence of RNA. (Right) The total volume of N condensates settled per micron squared area on the coverslip (mean ± SD, *n* = 20 with 2 technical replicates). A linear fit (solid line) reveals c_sat_ (± SE). **(B)** Images of the condensates formed in the presence of LD655-labeled WT N protein before or after phosphatase and kinase treatments in the presence of 10% PEG and 150 mM NaCl. **(C)** Images of the LD655-labeled truncated N protein mutants in the presence of 10% PEG and 150 mM NaCl. The protein concentration was set at 3 μM. Assays were performed in the absence of RNA. Data underlying this figure can be found in S1 Data. N, nucleocapsid; WT, wild-type.
(TIF)

S1 Table Cross-link fold changes with *p*-values less than 0.05 upon phase separation. The cross-link (xlink) position indicates the amino acid number of the N protein sequence. The K169-K65 peptide is phosphorylated at S176. N, nucleocapsid.
(DOCX)

S2 Table A compilation of the SARS-CoV-2 N protein phosphosites identified by MS. **(A)** The coverage map and phosphorylation sites for the N protein detected in proteomics experiments. Text in red indicates peptide regions that were not detected; green highlight indicates an unambiguous phosphorylation site; and yellow highlight indicates an ambiguous phosphorylation site. **(B)** Each site identified by our study is characterized as being unambiguously or ambiguously localized based on manual inspection of the product ion series. See Methods for the full protein sequence and a link to supporting evidence. S176 is a phosphorylation site identified on a cross-linked peptide. MS, mass spectrometry; N, nucleocapsid.
(DOCX)

S3 Table Proteins identified by MS in the high salt sample of N protein from human cells. **(A)** The proteins were ranked by SAF. These protein sequences were included in the CLMS search. Asterisks indicate exogenous proteins. **(B)** Cross-links between N and the G3BP1 and G3BP2 stress granule proteins identified in the qualitative CLMS experiment ranked by an SVM score. Values are frequency of detection (2 conditions with 2 replicates each). Xlink position indicates the amino acid number of the given protein sequence. SVM indicates the confidence that the cross-link is correctly identified. CLMS, cross-linking mass spectrometry; MS, mass spectrometry; N, nucleocapsid; SAF, spectral abundance factor; SVM, support vector machine.
(DOCX)

S4 Table Quantitative analysis of N condensates. The parameters of fitting to a dose–response equation in the presence and absence of polyC RNA in response to increasing concentrations of salt or nelfinavir mesylate. N, nucleocapsid.
(DOCX)

S1 Data Numerical data underlying main and Supporting information figures. Figs 1C–1H, 2C, 2D, 4D, 4F, 5B, 5C, and 6C–6E and S3B, S3C, S4D, S6B, S9B, S9D, S10B, S10D, S11B, S12A, S12B, S13B–S3D, S14B–S14D, and S15A Figs. Figs are presented in separate Excel sheets that are combined into a single Excel file. The numerical data include all replicates.
(XLSX)

S1 Raw Images Uncropped gel images underlying S1A, S1E and S1F, S2A, and S8AC Figs. (PDF)

## Abbreviations:

ACE2: angiotensin converting enzyme 2
BS3: bis(sulfosuccinimidyl) suberate
CLMS: cross-linking mass spectrometry
COVID-19: Coronavirus Disease 2019
CPE: cytopathic effect
CTD: carboxyl-terminal domain
EMCCD: electron-multiplying CCD
ERGIC: ER-Golgi intermediate compartment
FBS: fetal bovine serum
FDA: Food and Drug Administration
FDR: false discovery rate
FISH: fluorescence in situ hybridization
FRAP: fluorescence recovery after photobleaching
HEK293: human embryonic kidney
HiLO: highly inclined and laminated optical sheet
hpi: hours postinfection
IDR: intrinsically disordered region
LC–MS: liquid chromatography–mass spectrometry
LLPS: liquid–liquid phase separation
M: membrane
MAVS: mitochondrial antiviral-signaling protein
MOI: multiplicity of infection
MS: mass spectrometry
N: nucleocapsid
NTD: N-terminal domain
PLD: prion-like domain
PS: penicillin-streptomycin
RTC: replication transcription complex
S: spike
SAF: spectral abundance factor
SARS-CoV-2: Severe Acute Respiratory Syndrome Coronavirus 2
SEC: size exclusion chromatography
SLIP: site localization in peptide
SR: serine/arginine-rich
TCID: tissue culture infectious dose
TIRF: total internal reflection fluorescence
UCSD: University of California San Diego
UTR: untranslated region
VLP: viral-like particle
vRNP: viral ribonucleoprotein
WT: wild-type
XIC: extracted precursor ion chromatogram
