# Supplementary figures and images for: SARS-CoV-2 nucleocapsid protein forms condensates with viral genomic RNA
Source: PLoS Biol. 2021 Oct 11;19(10):e3001425. doi: 10.1371/journal.pbio.3001425 (PMC8553124; doi:10.1371/journal.pbio.3001425)

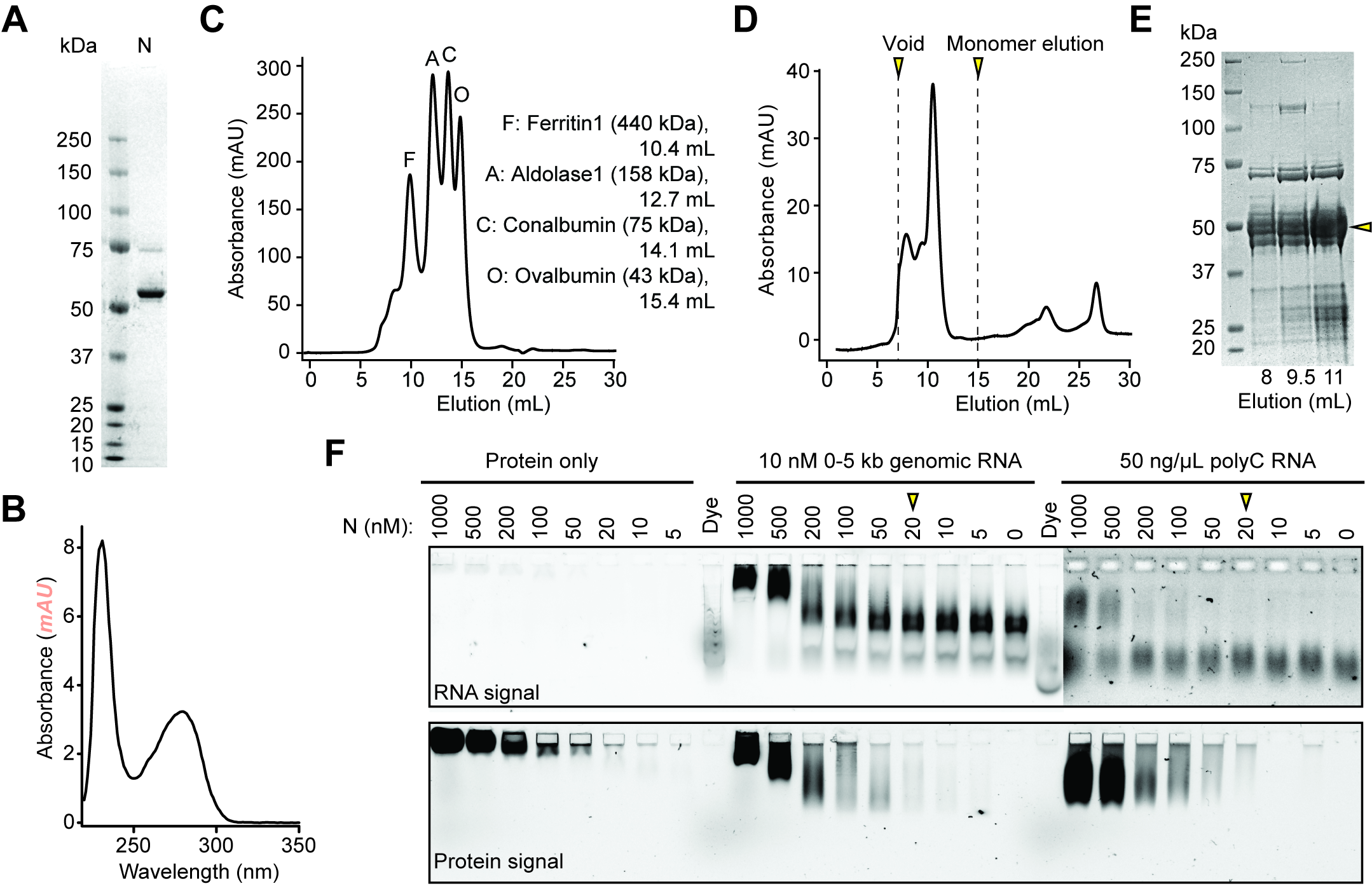

Supplement: S1 Fig — (A) The coomassie-stained denaturing gel of the N protein purified from affinity chromatography. (B) UV absorbance of the N protein purified from affinity chromatography shows no evidence for the presence of contaminating nucleic acids. (C) UV absorbance of protein standards eluting from a gel filtration column. (D) UV absorbance of the N protein eluting from a gel filtration column. Arrows mark the void volume and expected elution volume for an N monomer. (E) The coomassie-stained denaturing gel of the eluents from the gel filtration column. The yellow arrowhead shows the expected molecular weight of full-length N protein. (F) EMSA using no RNA, 10 nM 0–5 kb viral RNA, and 50 ng/μL polyC RNA and decreasing concentration of N protein. The protein was labeled with LD655. RNA was labeled with Cy3. Arrowheads indicate the minimum protein concentration for each condition with a noticeable signal in the protein gel. EMSA, electrophoresis mobility shift assay; N, nucleocapsid; WT, wild-type. (TIF) [file pbio.3001425.s001.tif]

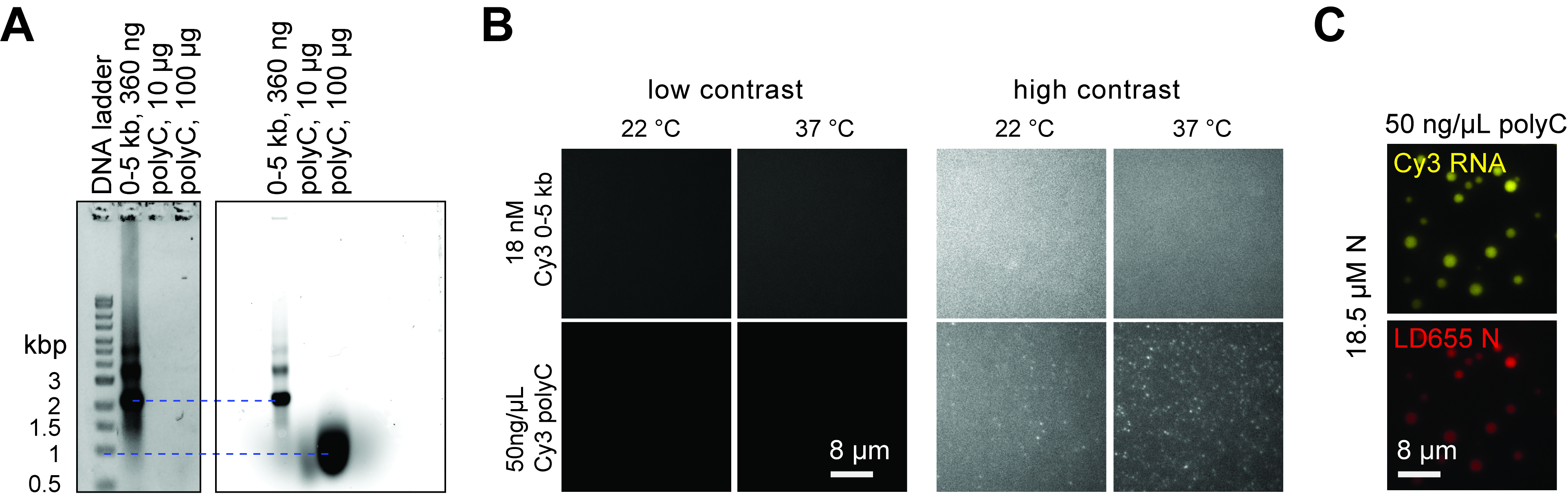

Supplement: S2 Fig — (A) An agarose gel picture of 0–5 kb viral RNA and polyC RNA substrates. The gel was stained with GelRed (left), and the RNA substrates were labeled with Cy3 (right). The ladder corresponds to the length of double-stranded DNA. Estimated lengths of 0–5 kb viral RNA and polyC RNA are 5 kb and 2 kb, respectively. (B) Representative pictures show that 18 nM 0–5 kb viral RNA and 50 ng/μL polyC RNA do not form condensates in the absence of N protein. The assay was performed in 150 mM NaCl. (C) Two-color imaging shows colocalization of LD655-labeled N protein and Cy3-labeled polyC RNA in condensates. N, nucleocapsid. (TIF) [file pbio.3001425.s002.tif]

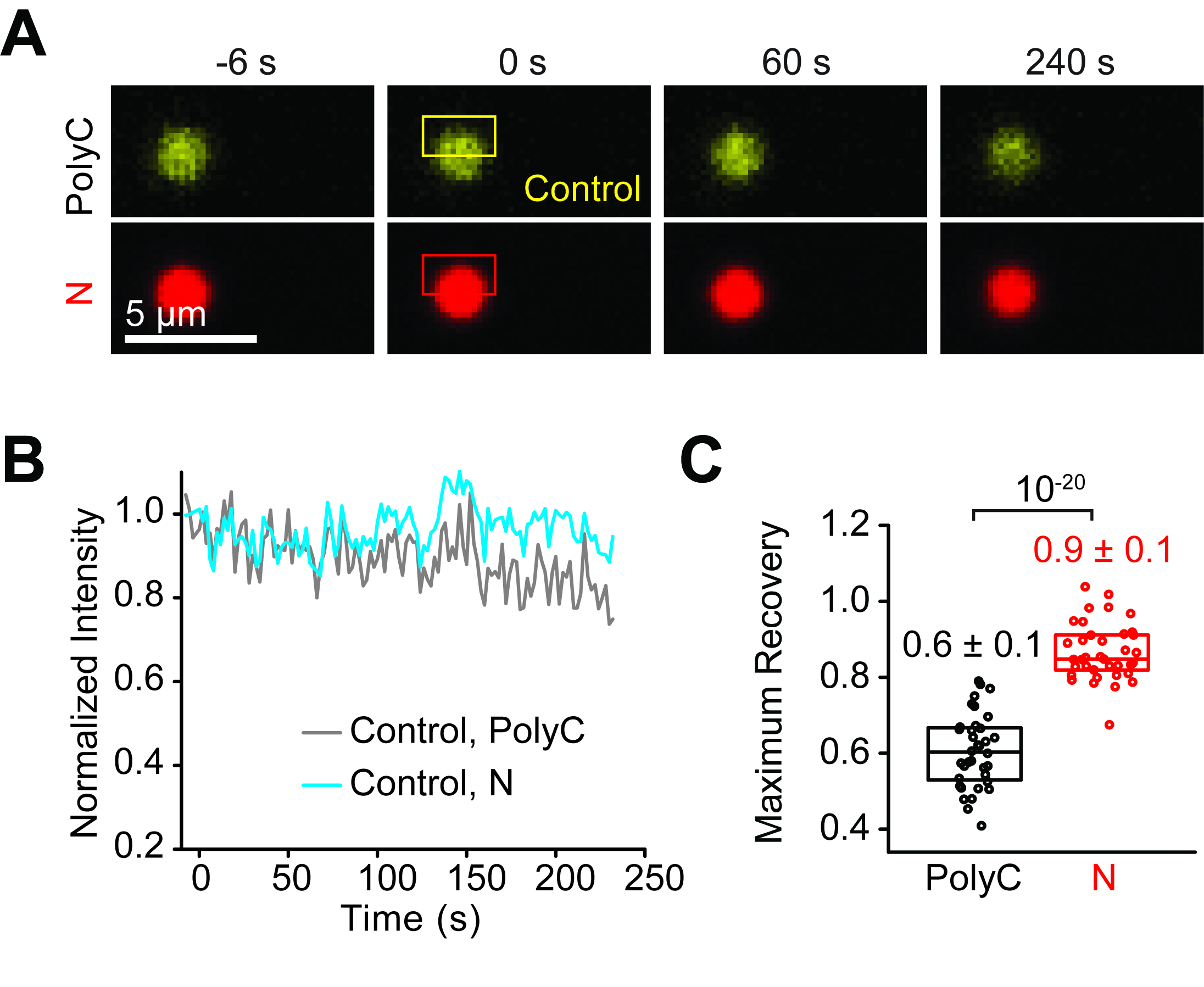

Supplement: S3 Fig — (A) Control experiments show changes in the fluorescent signal of N and polyC without photobleaching. (B) The changes in the integrated fluorescent intensities of regions highlighted with yellow and red rectangles in (A). (C) The maximum fractional recovery of N and polyC in condensates after photobleaching (n = 36, mean ± SD). The center and edges of the box represent the median with the first and third quartiles. The p-value was calculated from a 2-tailed t test. Data underlying this figure can be found in S1 Data. FRAP, fluorescence recovery after photobleaching; N, nucleocapsid. (TIF) [file pbio.3001425.s003.tif]

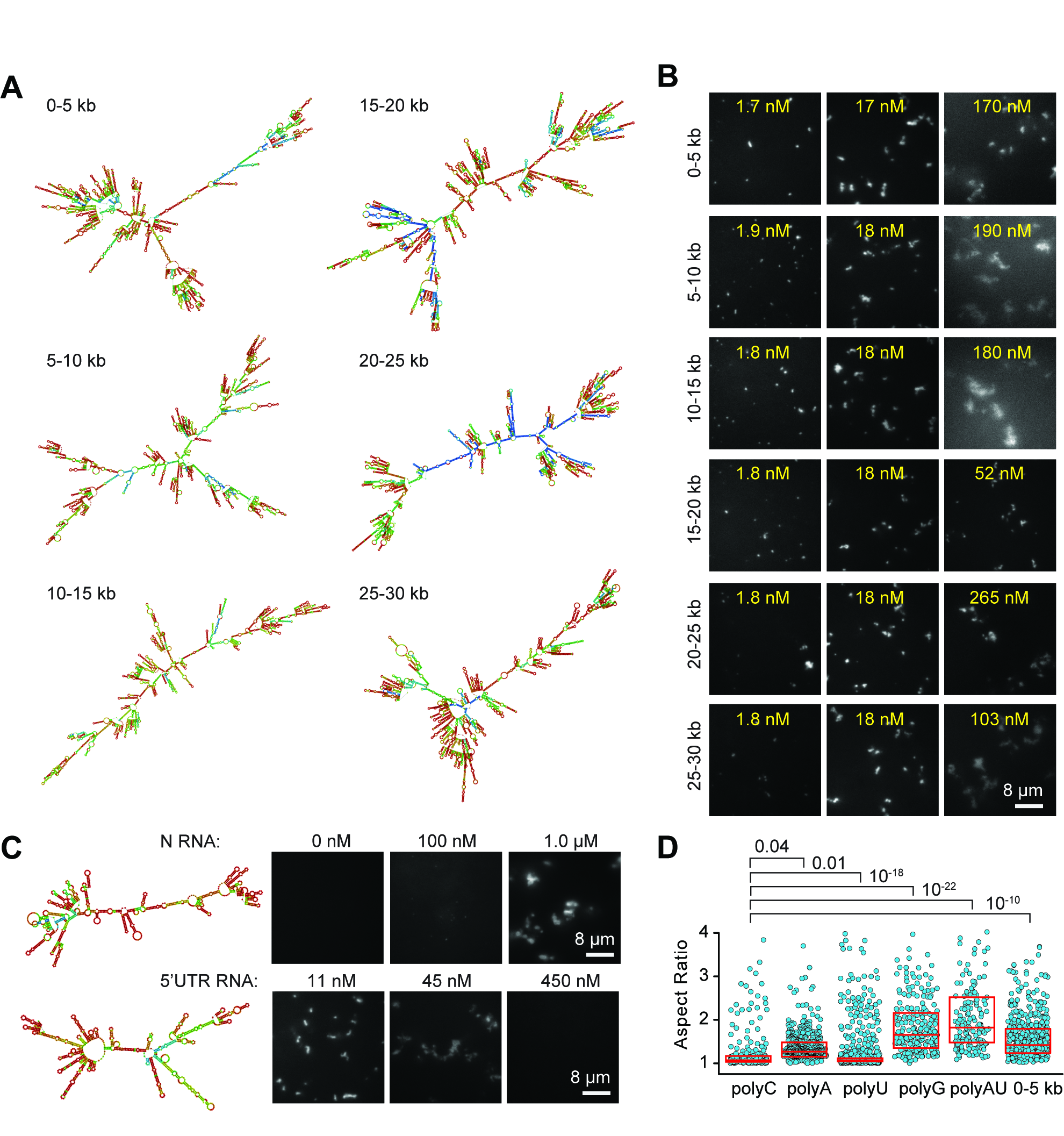

Supplement: S4 Fig — (A) Structure prediction of each section of SARS-CoV-2 genomic RNA. (B) The formation of asymmetric N condensates under different viral RNA concentrations. The N protein concentration was set to 18.5 μM. (C) Structure prediction of viral RNA (left) and the formation of asymmetric N condensates under different RNA concentrations (right). The N RNA is the 1.3 kb long genomic RNA fragment that encodes the SARS-CoV-2 N protein. The 5′ UTR RNA is the first 1,000 bases of the SARS-CoV-2 genome. The N protein concentration was set to 18.5 μM. (D) The distribution of aspect ratios of individual condensates formed with different RNA substrates. The N protein concentration was set to 18.5 μM, and RNA concentration was set to 50 ng/μL for RNA homopolymers and 18 nM for 0–5 kb viral RNA. The center and edges of the box represent the median with the first and third quartiles. p-Values are calculated from 2-tailed t tests. Data underlying this figure can be found in S1 Data. IVT, in vitro transcribed; N, nucleocapsid; SARS-CoV-2, Severe Acute Respiratory Syndrome Coronavirus 2; UTR, untranslated region. (TIF) [file pbio.3001425.s004.tif]

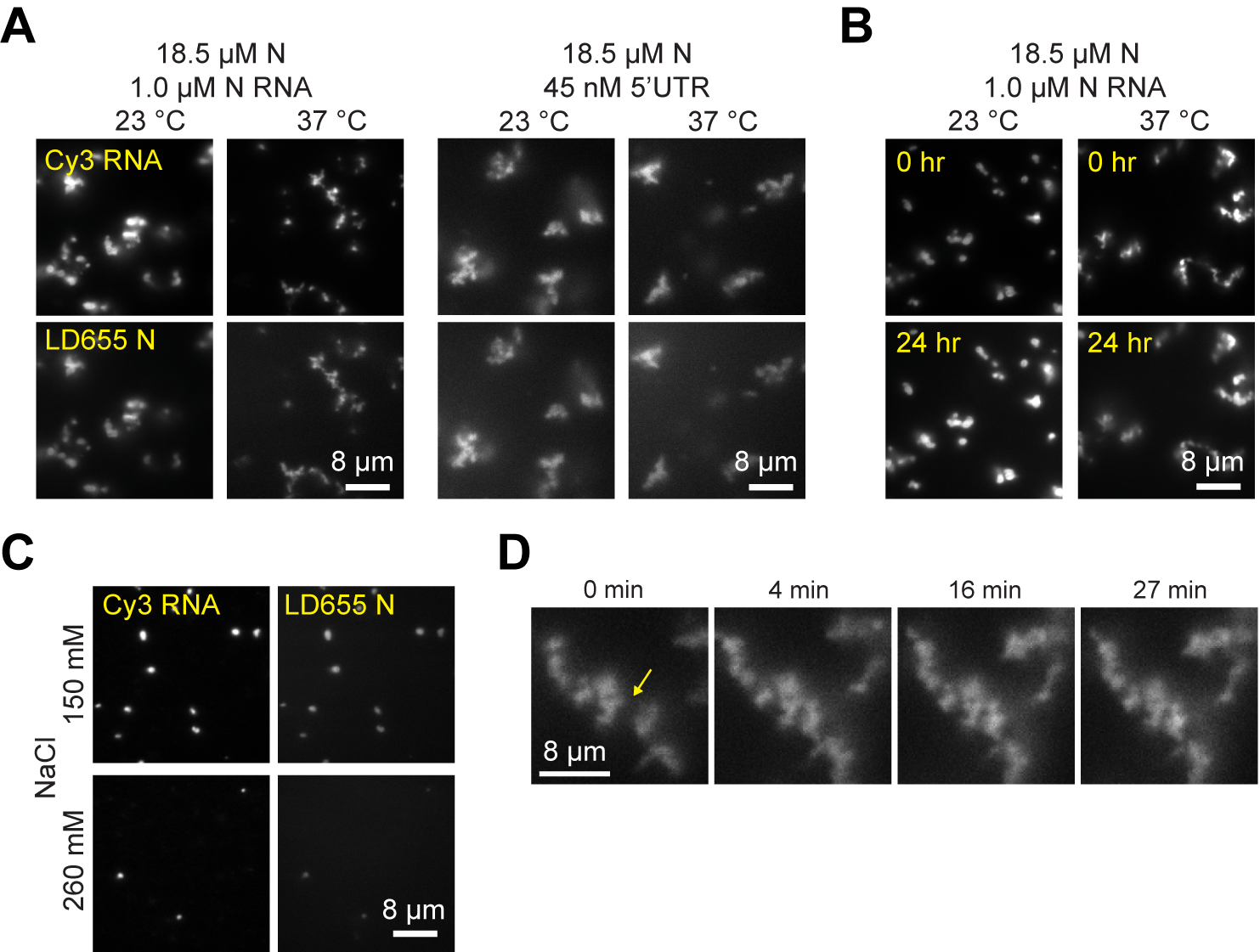

Supplement: S5 Fig — (A) Condensates formed by the N protein with in vitro transcribed N RNA or the 5′ UTR of the viral RNA are not sensitive to an increase of temperature to 37°C. (B) Condensates formed by the N protein with 5′ UTR of viral RNA do not change shape over 24 hours. (C) Condensates formed by 11.5 μM N protein and 36 nM 5′ UTR RNA are dissolved in the presence of 260 mM NaCl. (D) Condensates formed by 18.5 μM N protein and 18 nM 0–5 kb viral RNA do not fuse after contact (yellow arrow). N, nucleocapsid; UTR, untranslated region. (TIF) [file pbio.3001425.s005.tif]

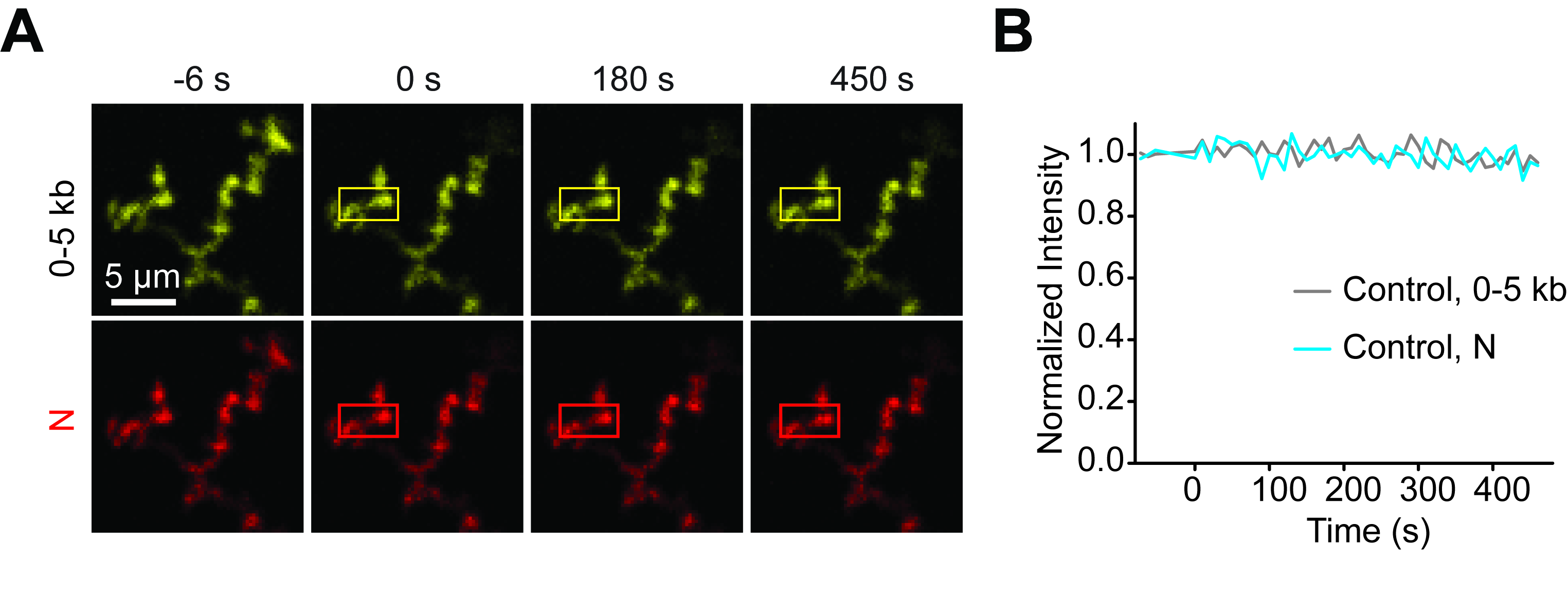

Supplement: S6 Fig — (A) Control experiments show snapshots of LD655-labeled N and Cy3-labeled 0–5 kb viral RNA without photobleaching. (B) The changes in the integrated fluorescent intensities of the regions that are highlighted with yellow and red rectangles in (A). Data underlying this figure can be found in S1 Data. FRAP, fluorescence recovery after photobleaching; N, nucleocapsid. (TIF) [file pbio.3001425.s006.tif]

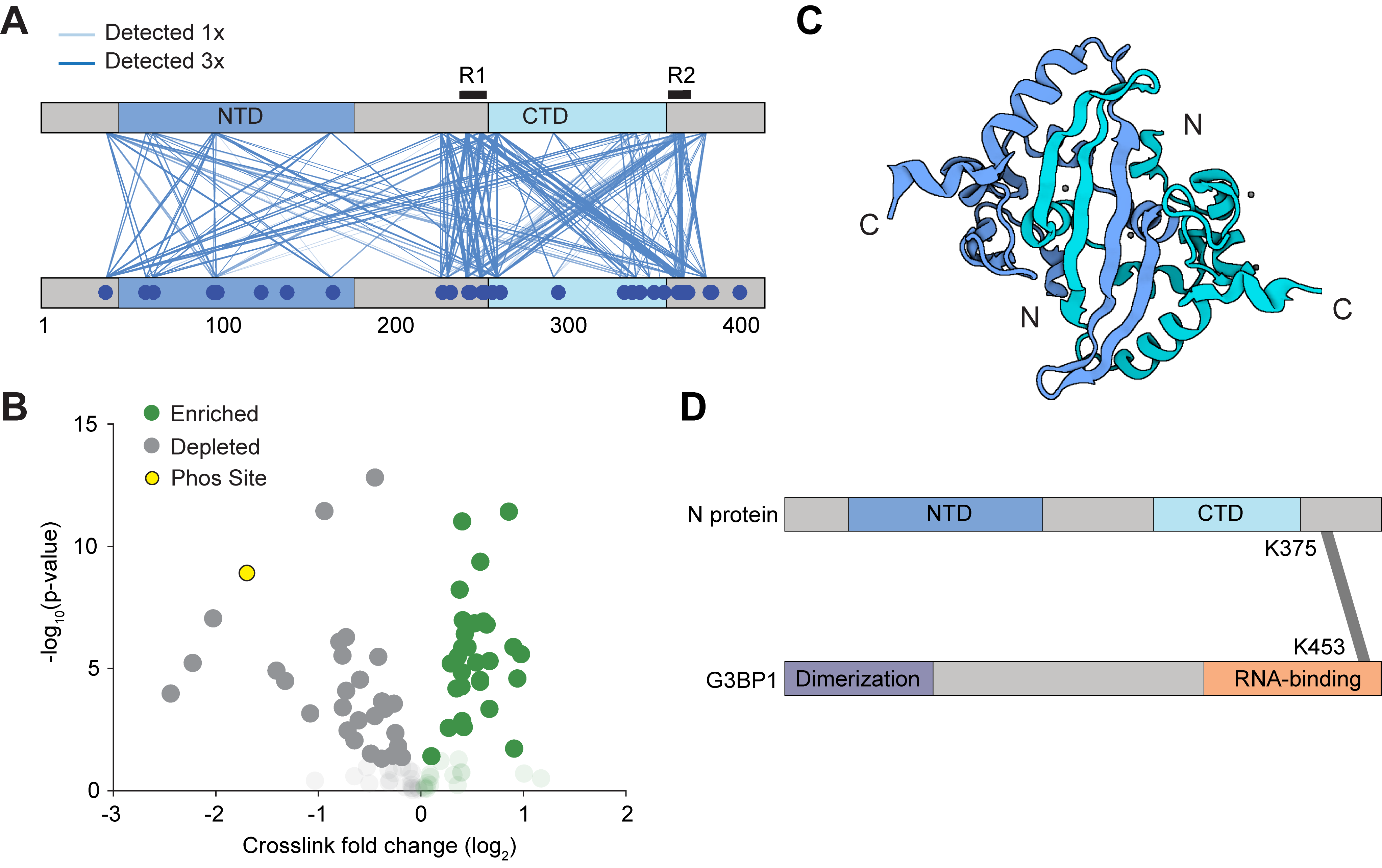

Supplement: S7 Fig — (A) In an initial qualitative experiment, pairwise interactions were detected by CLMS at 300 mM salt. Blue dots depict the positions of lysine residues. Lines depict a unique cross-link detected. The regions of N protein interactions flank the CTD. (B) Volcano plot of the quantitative CLMS data comparing the condensate and no condensate condition. Opaque data points have a p-value below 0.05, and transparent data points have a p-value greater than 0.05. Green represents unique cross-links that are enriched in the condensate condition. The yellow markers represent the K169-K65 and K169 (phosS176)-K65 cross-links. (C) The structure of the CTD of the SARS-CoV-2 N protein was plotted with BioRender (PDB 6WJI [89]). Because the N terminus and carboxyl terminus of the protomers are positioned away from each other, R1 and R2 within the same dimer are unlikely to interact with each other. (D) MS identified that the RNA binding domain of the stress granule protein G3BP1 interacts with the R2 region of N. Data underlying this figure can be found in S1 Table. CLMS, cross-linking mass spectrometry; CTD, carboxyl-terminal domain; MS, mass spectrometry; N, nucleocapsid; SARS-CoV-2, Severe Acute Respiratory Syndrome Coronavirus 2. (TIF) [file pbio.3001425.s007.tif]

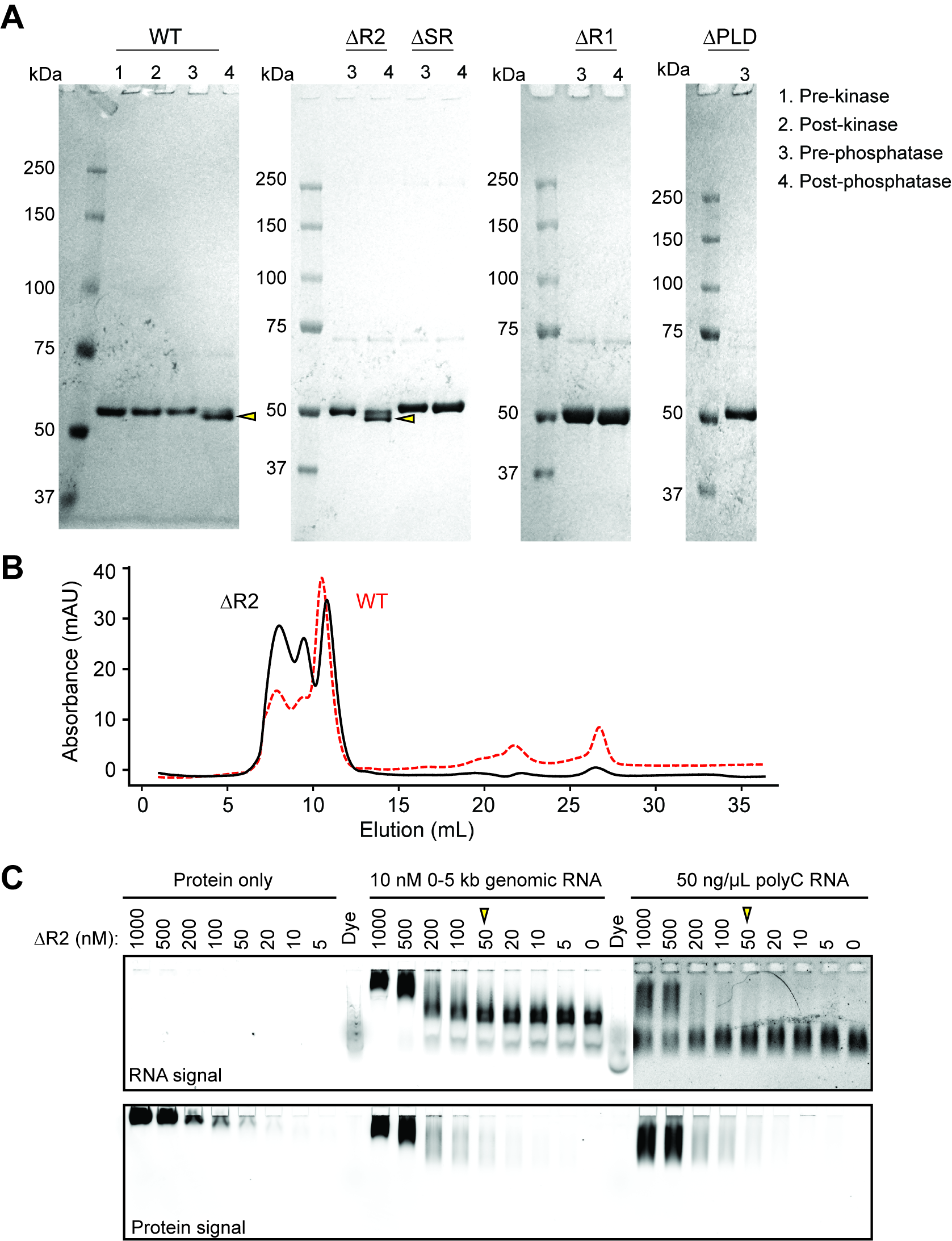

Supplement: S8 Fig — (A) Denaturing gel pictures of purified WT and deletion mutants of N protein in the presence and absence of kinase and phosphatase treatment (see Methods). The gels were stained with Coomassie. Yellow arrowheads highlight a reduction in molecular weight upon treatment with λ phosphatase. (B) UV absorbance of the ΔR2 mutant eluting from a gel filtration column. UV absorbance of WT N under the same experimental conditions is shown in a dashed red curve for comparison. (C) EMSA gels using no RNA, 10 nM 0–5 kb viral RNA, or 50 ng/μL polyC RNA and decreasing concentration of the ΔR2 mutant. The protein was labeled with LD655. RNA was labeled with Cy3. Arrows indicate the minimum protein concentration for each condition with a noticeable signal in the protein gel. EMSA, electrophoresis mobility shift assay; MS, mass spectrometry; N, nucleocapsid; WT, wild-type. (TIF) [file pbio.3001425.s008.tif]

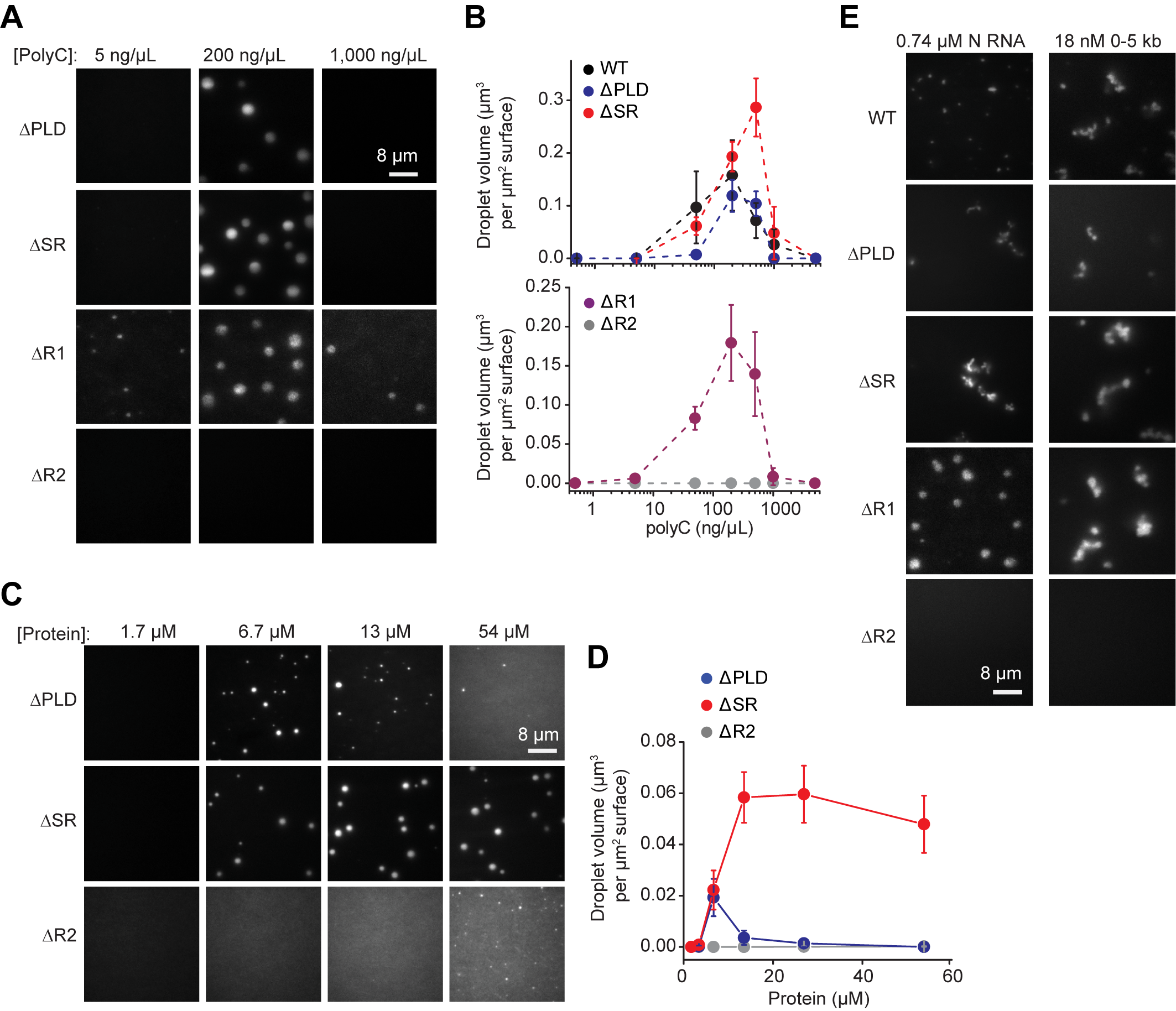

Supplement: S9 Fig — (A) Example pictures show that the N deletion mutants, except ΔR2, form spherical condensates with polyC RNA under different RNA concentrations. The N protein concentration was set to 18.5 μM. (B) The total volume of N-RNA condensates settled per micron squared area on the coverslip (mean ± SD; n = 20, 2 technical replicates) exhibits a reentrant behavior under an increasing RNA concentration. (C) Example pictures show that the N deletion mutants, except ΔR2, form spherical condensates with polyC RNA under different protein concentrations. The polyC RNA concentration was set to 50 ng/μL. (D) The total volume of N-RNA condensates settled per micron squared area on the coverslip (mean ± SD; n = 20, 2 technical replicates) under an increasing protein concentration. (E) Phase separation of truncated N protein constructs with 0.74 μM in vitro transcribed N RNA or 18 nM 0–5 kb viral RNA. The protein concentration was set to 18.5 μM. Data underlying this figure can be found in S1 Data. N, nucleocapsid. (TIF) [file pbio.3001425.s009.tif]

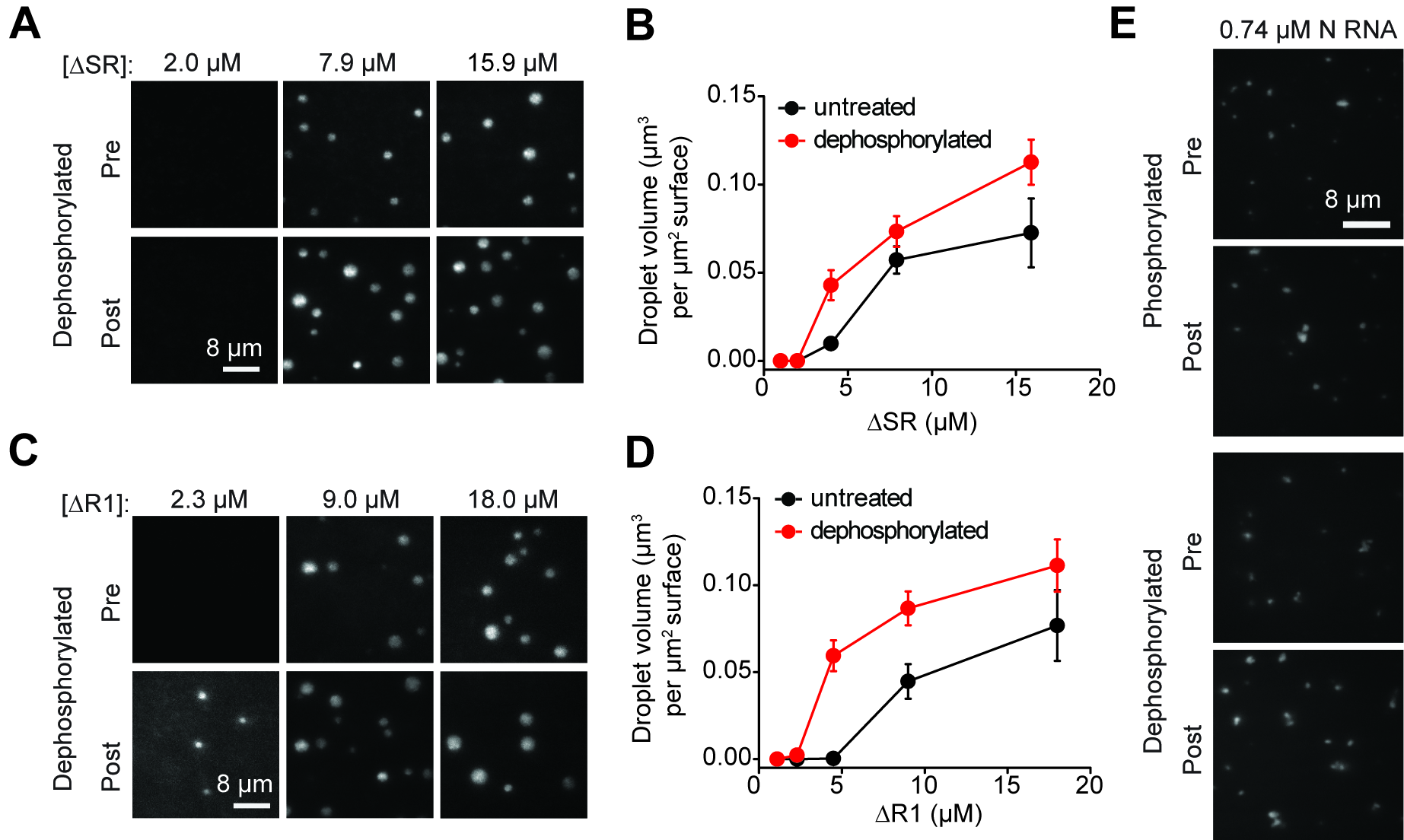

Supplement: S10 Fig — (A) Images of condensates formed by untreated and dephosphorylated ΔSR in the presence of 50 ng/μL polyC RNA. (B) The total volume of the condensates settled per micron squared area on the coverslip as a function of ΔSR concentration (mean ± SD, n = 20 with 2 technical replicates). (C) Images of condensates formed by untreated and dephosphorylated ΔR1 in the presence of 50 ng/μL polyC RNA. (D) The total volume of the condensates settled per micron squared area on the coverslip as a function of ΔR1 concentration (mean ± SD, n = 20 with 2 technical replicates). (E) Full-length N protein forms asymmetric condensates with 0.74 μM in vitro transcribed N RNA before and after phosphorylation and dephosphorylation. The protein concentration was set to 35 μM. Data underlying this figure can be found in S1 Data. N, nucleocapsid. (TIF) [file pbio.3001425.s010.tif]

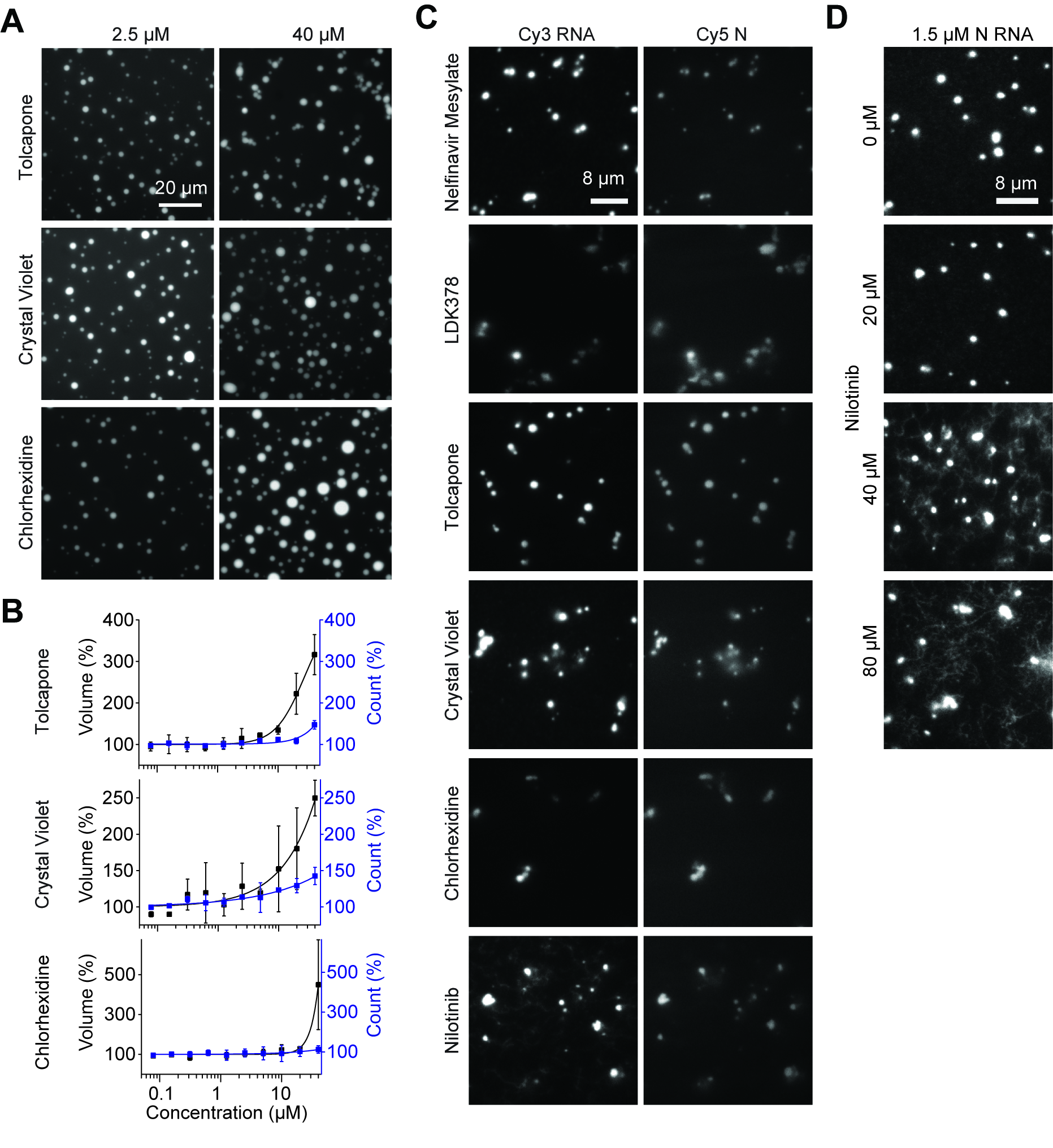

Supplement: S11 Fig — (A) Example pictures show phase separation of 7.8 μM N protein and 50 ng/μL polyC RNA at different drug concentrations. (B) The percent change on the number (blue) and total volume (black) of N-polyC condensates settled per micron squared area on the coverslip under different drug concentrations (mean ± SD, n = 8 with 2 technical replicates). (C) Example pictures show phase separation of 57.6 μM LD655-labeled N protein and 1.5 μM Cy3-labeled in vitro transcribed N RNA in the presence of different drug treatments. The concentration of drugs was set to 40 μM. (D) Condensates formed in the presence of 57.6 μM N protein and 1.5 μM N RNA form thread-like filaments at high nilotinib concentrations. Data underlying this figure can be found in S1 Data. N, nucleocapsid. (TIF) [file pbio.3001425.s011.tif]

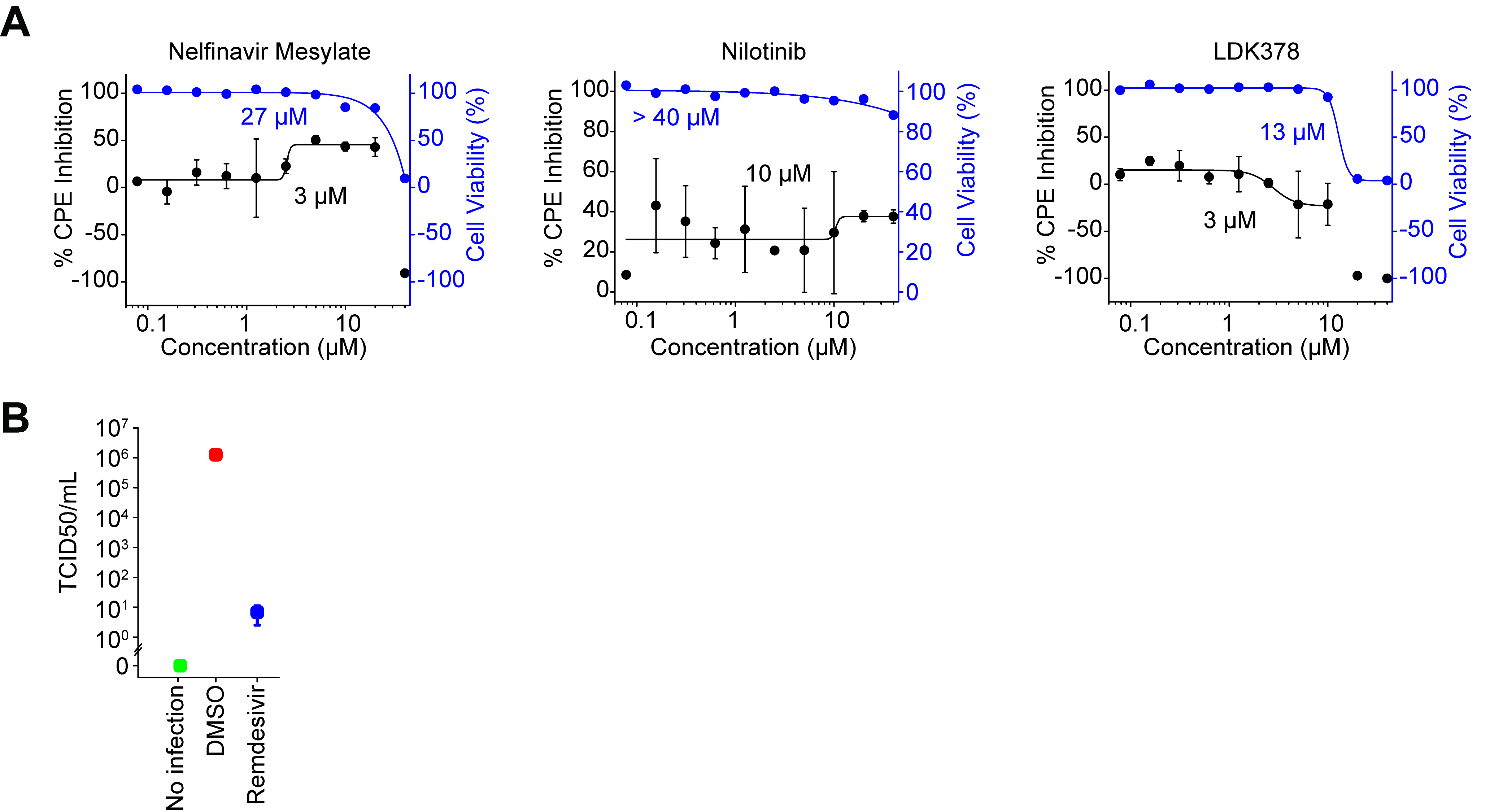

Supplement: S12 Fig — (A) Percent CPE inhibition (black, mean ± SD, 2 technical replicates) and cell viability (blue) of SARS-CoV-2–infected Calu-3 cells treated with serial dilutions of drugs. Solid curves represent a fit to a dose–response equation to determine EC50. (B) Viral titer in uninfected and SARS-CoV-2–infected Vero-E6 cells as measured by a TCID50 assay. Infected cells were treated with DMSO (negative control) and 50 μM remdesivir (positive control). Data underlying this figure can be found in S1 Data. CPE, cytopathic effect; SARS-CoV-2, Severe Acute Respiratory Syndrome Coronavirus 2; TCID, tissue culture infectious dose. (TIF) [file pbio.3001425.s012.tif]

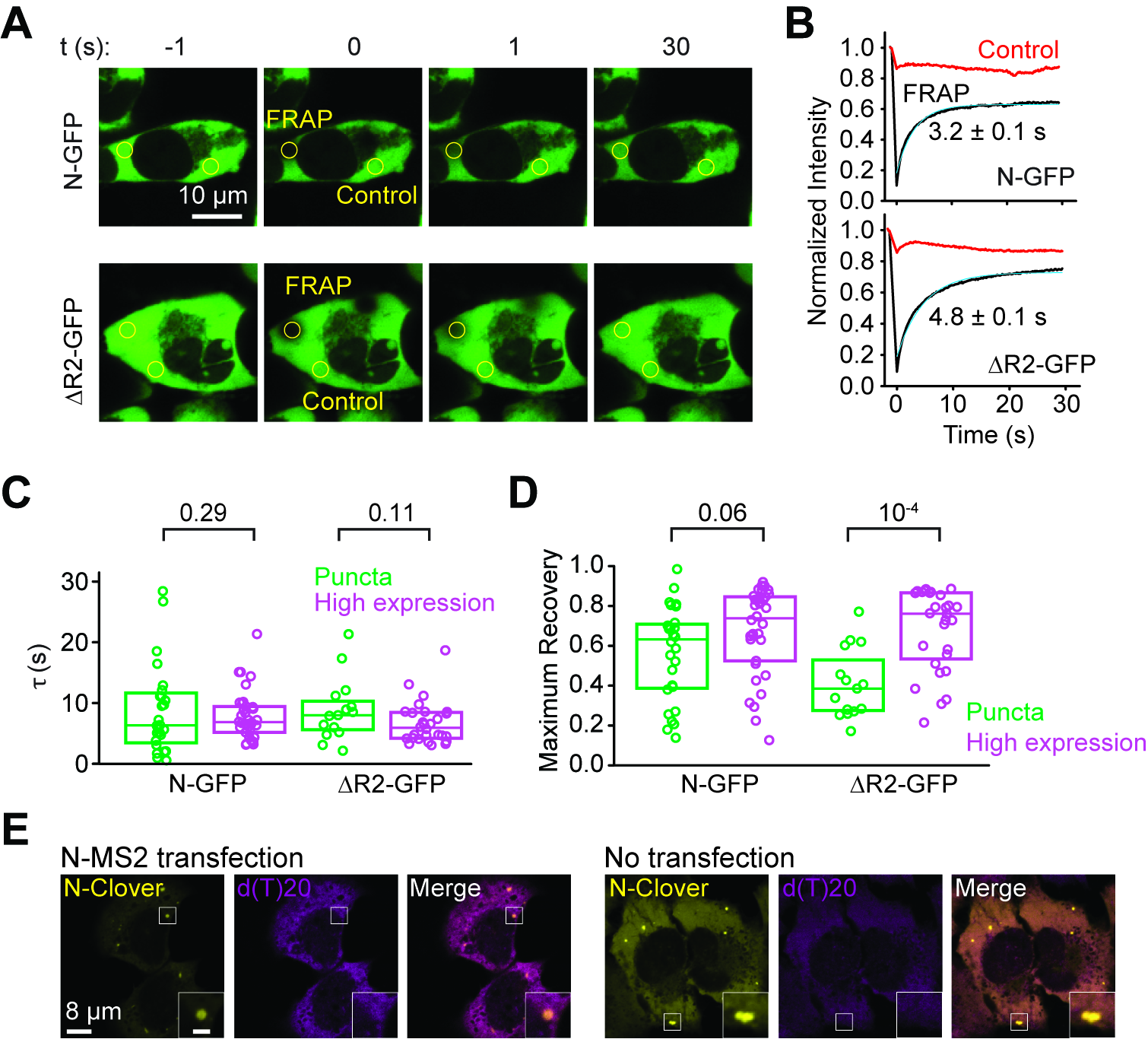

Supplement: S13 Fig — (A) Representative FRAP imaging of HEK293T cells with high expression of N-GFP or ΔR2-GFP. Circles show the photobleached and control (not bleached) regions. (B) Fluorescence recovery signals of the N protein in the bleached versus the control regions. The solid curve represents a single exponential fit to reveal the recovery lifetime (τ, ±95% confidence interval). (C) The distribution of fluorescence recovery lifetimes of cells expressing N-GFP or ΔR2-GFP and exhibiting either puncta or high expression (from left to right, n = 28, 34, 15, and 28). The center and edges of the box represent the median with the first and third quartiles. The p-values were calculated from a 2-tailed t test. (D) The maximum fractional recovery after photobleaching of cells expressing N-GFP or ΔR2-GFP and exhibiting either puncta or high expression (from left to right, n = 28, 34, 15, and 28). The center and edges of the box represent the median with the first and third quartiles. The p-values were calculated from a 2-tailed t test. (E) U2OS cells stably expressing N-Clover form condensates in the cytoplasm. Cy3-d(T)20 FISH probe targeting the polyA tails of RNA transcripts is uniformly distributed in cells exhibiting N condensates with (left) or without (right) expression of N-MS2 (N = 40 cells, 2 technical replicates). Inset scale bar is 2 μm. Data underlying this figure can be found in S1 Data. FRAP, fluorescence recovery after photobleaching; N, nucleocapsid. (TIF) [file pbio.3001425.s013.tif]

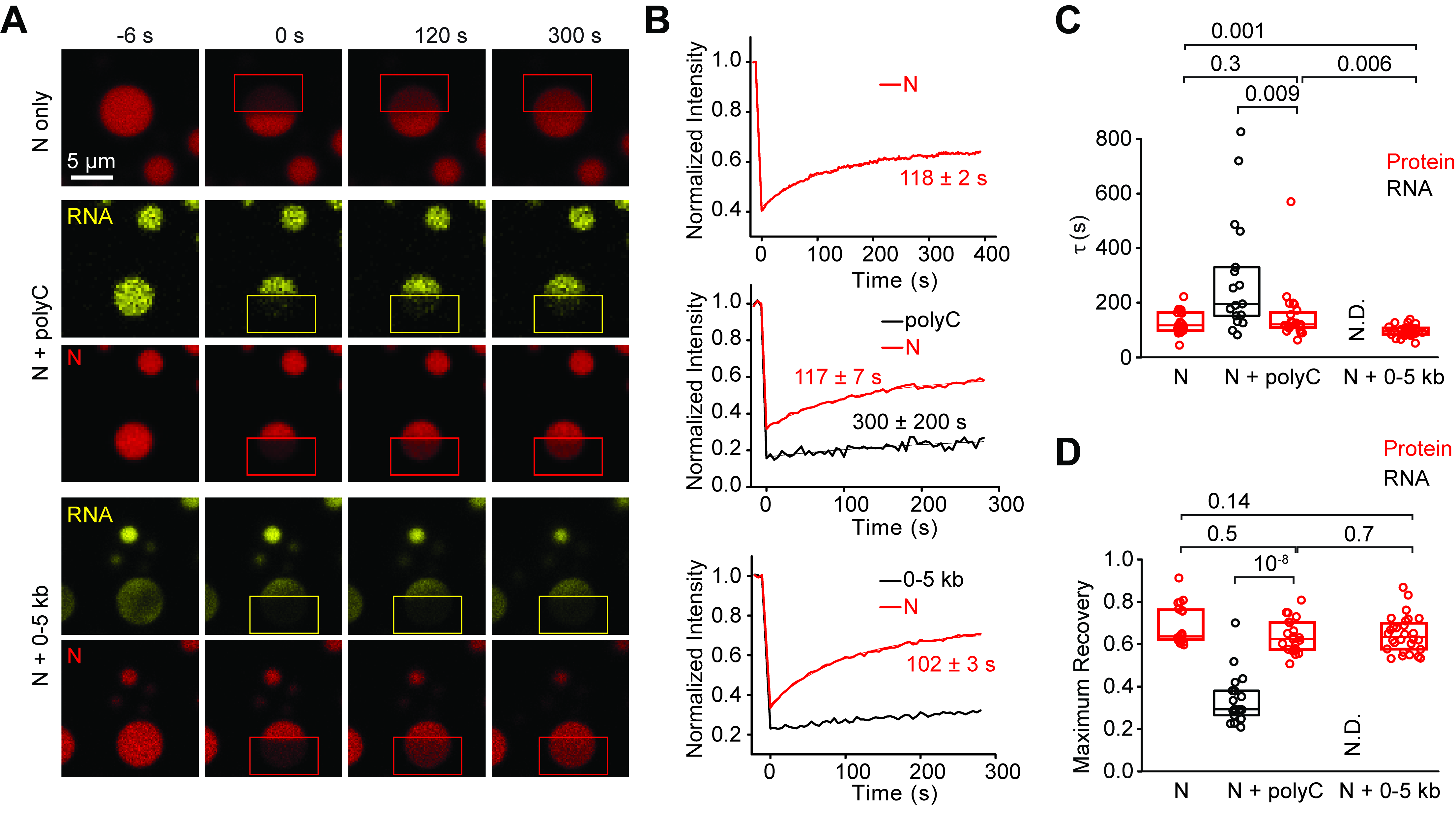

Supplement: S14 Fig — (A) Representative FRAP imaging of N protein only, or in the presence of polyC or 0–5 kb viral RNA. The concentrations of N, polyC, and 0–5 kb viral RNA were kept at 24 μM, 50 ng/μL, and 18 nM, respectively. Rectangles highlight the photobleached area. (B) Fluorescence recovery signals of the N protein and RNA in the bleached versus the control regions. Solid curves represent a single exponential fit to reveal the recovery lifetime (τ, ±95% confidence interval). (C) The distribution of fluorescence recovery lifetimes of the condensates (from left to right, n = 20, 17, 23, 28, and 28). The center and edges of the box represent the median with the first and third quartiles. The p-values were calculated from a 2-tailed t test. (D) The maximum fractional recovery after photobleaching (from left to right, n = 20, 17, 23, 28, and 28). The center and edges of the box represent the median with the first and third quartiles. The p-values were calculated from a 2-tailed t test. Data underlying this figure can be found in S1 Data. FRAP, fluorescence recovery after photobleaching; N, nucleocapsid. (TIF) [file pbio.3001425.s014.tif]

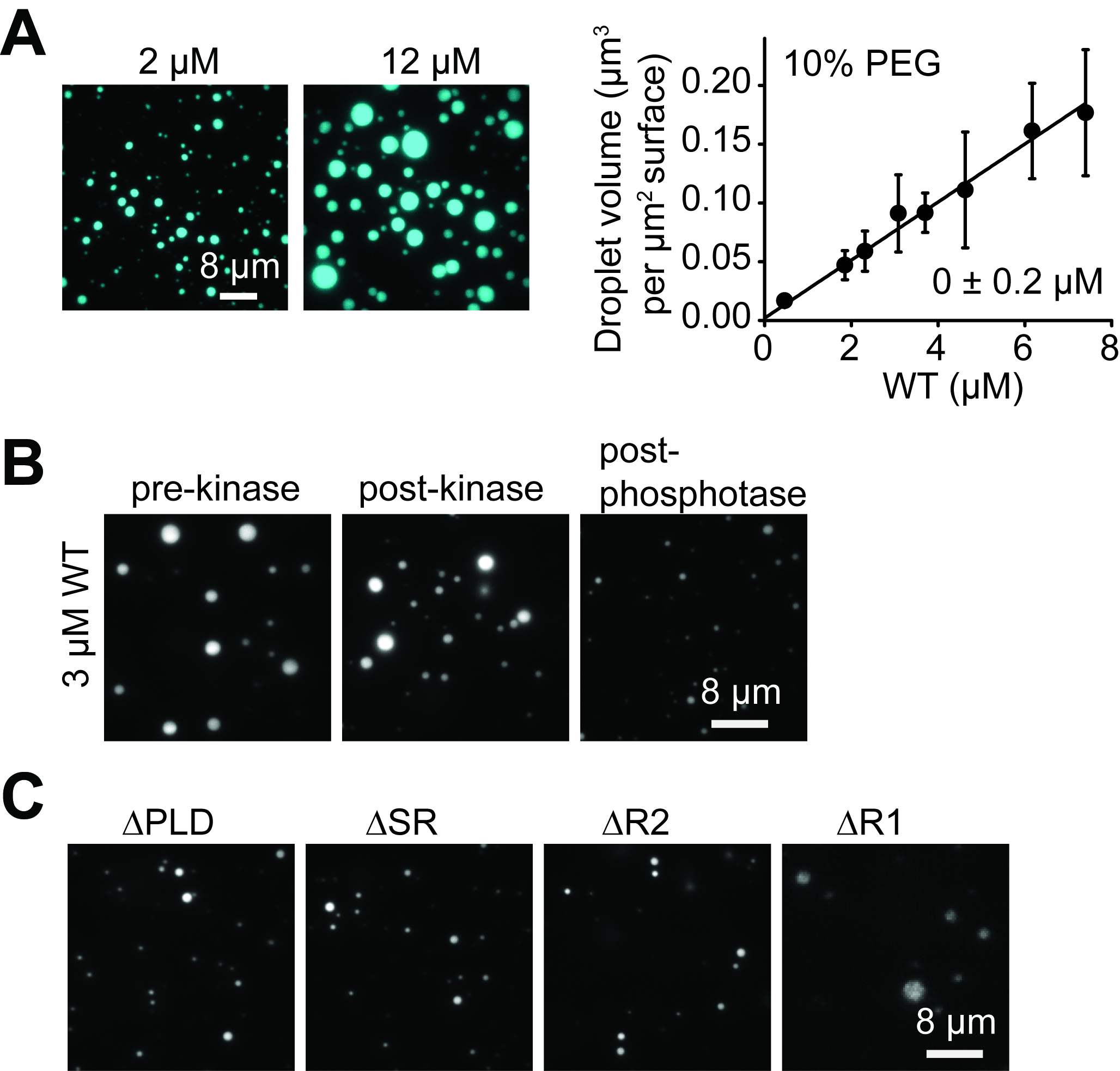

Supplement: S15 Fig — (A) (Left) Images of the LD655-labeled N protein in the presence of 10% PEG and 150 mM NaCl. The assays were performed in the absence of RNA. (Right) The total volume of N condensates settled per micron squared area on the coverslip (mean ± SD, n = 20 with 2 technical replicates). A linear fit (solid line) reveals csat (± SE). (B) Images of the condensates formed in the presence of LD655-labeled WT N protein before or after phosphatase and kinase treatments in the presence of 10% PEG and 150 mM NaCl. (C) Images of the LD655-labeled truncated N protein mutants in the presence of 10% PEG and 150 mM NaCl. The protein concentration was set at 3 μM. Assays were performed in the absence of RNA. Data underlying this figure can be found in S1 Data. N, nucleocapsid; WT, wild-type. (TIF) [file pbio.3001425.s015.tif]

S1A Fig

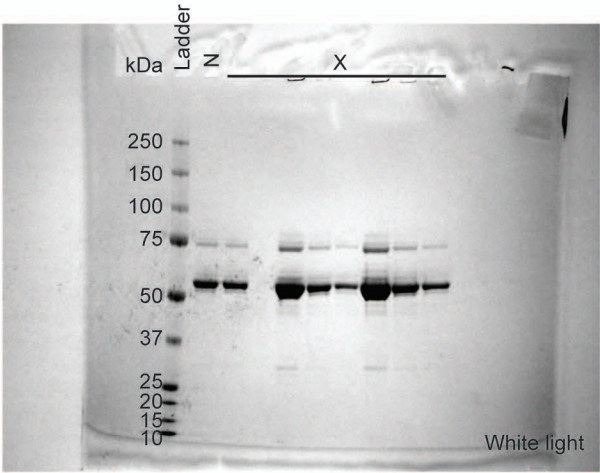

S1E Fig

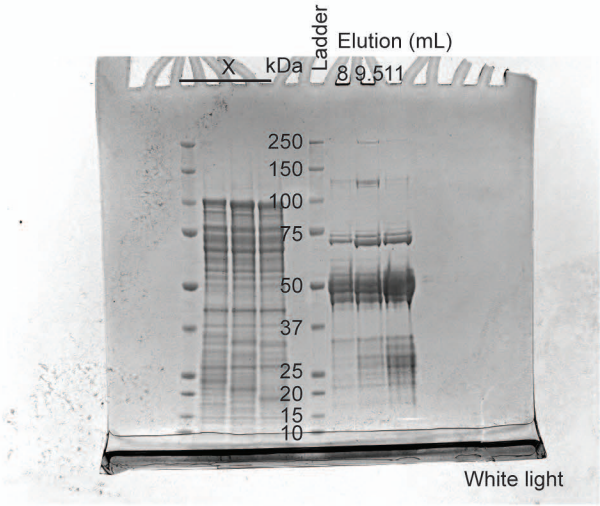

**S1F Fig**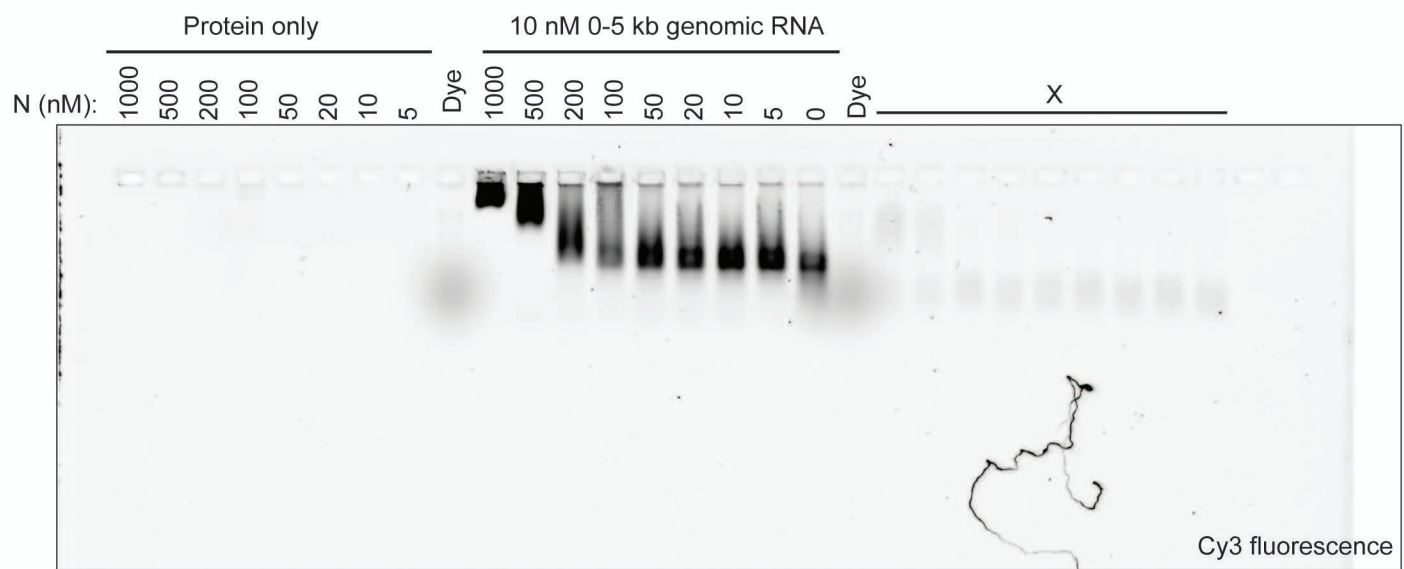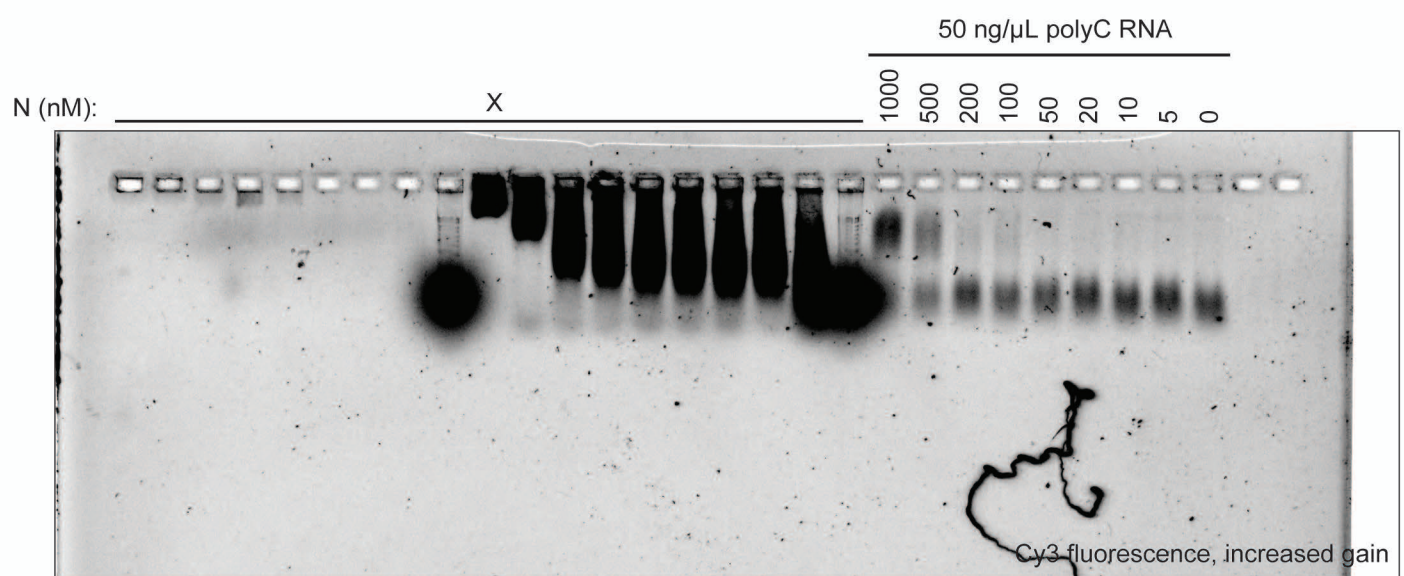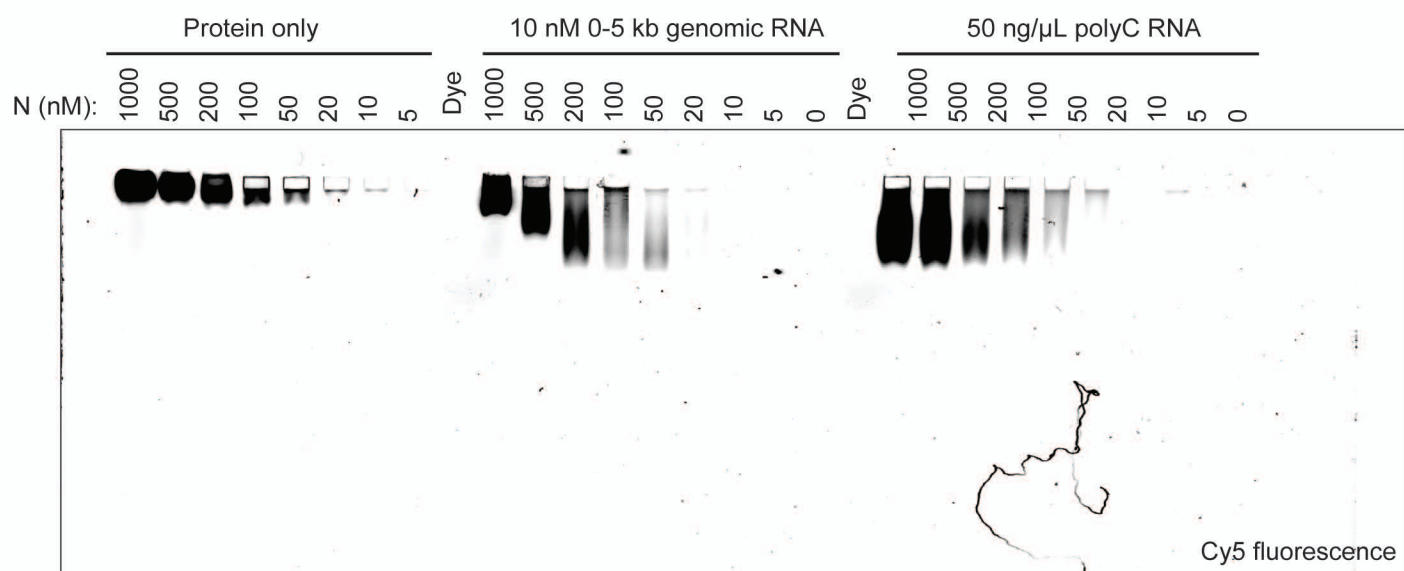

S2A Fig

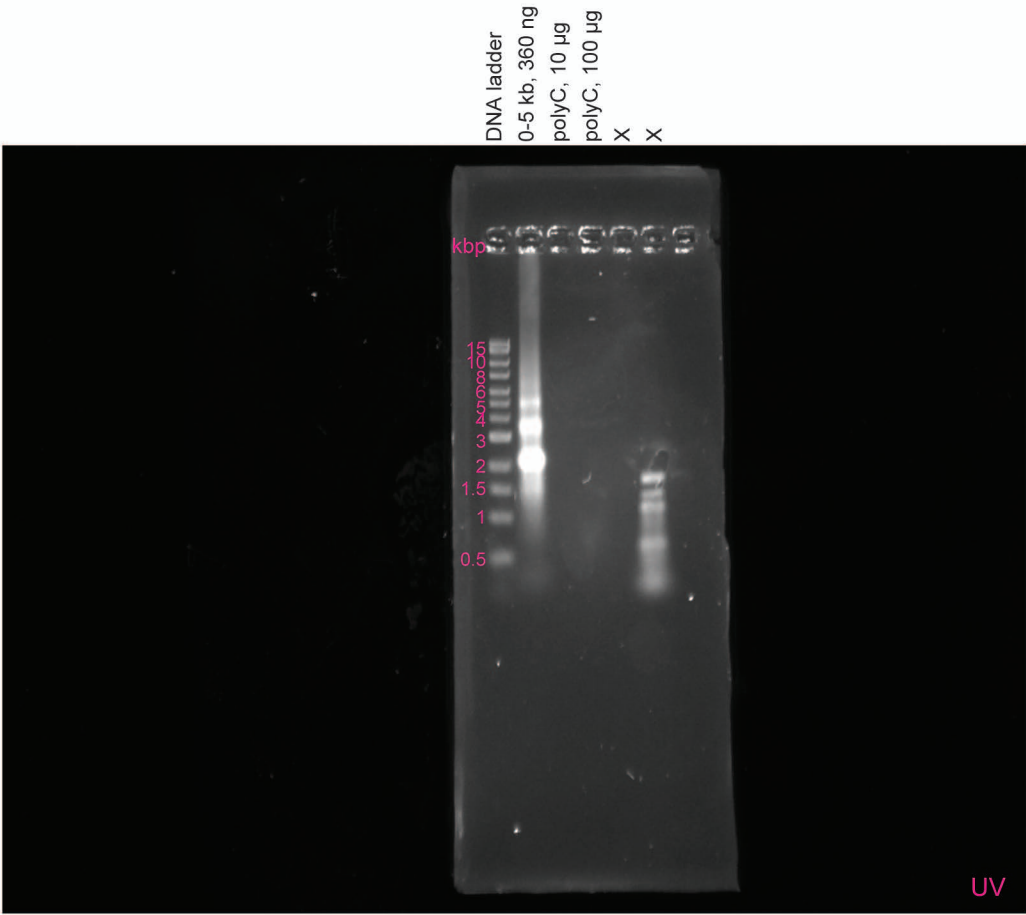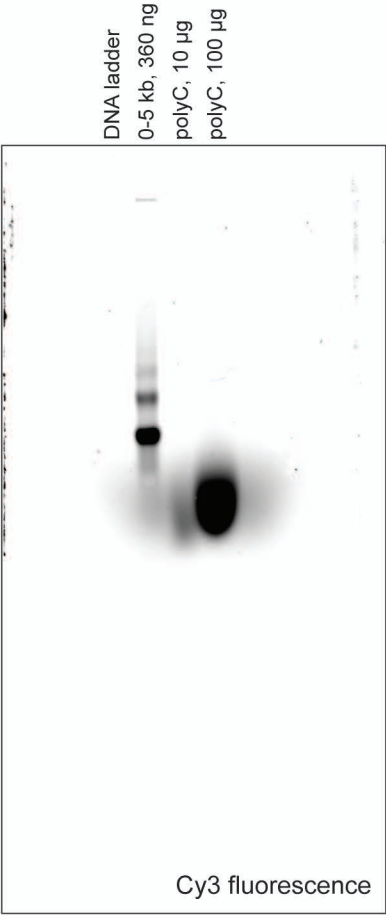

S8A Fig

- 1. Pre-kinase
- 2. Post-kinase
- 3. Pre-phosphatase
- 4. Post-phosphatase

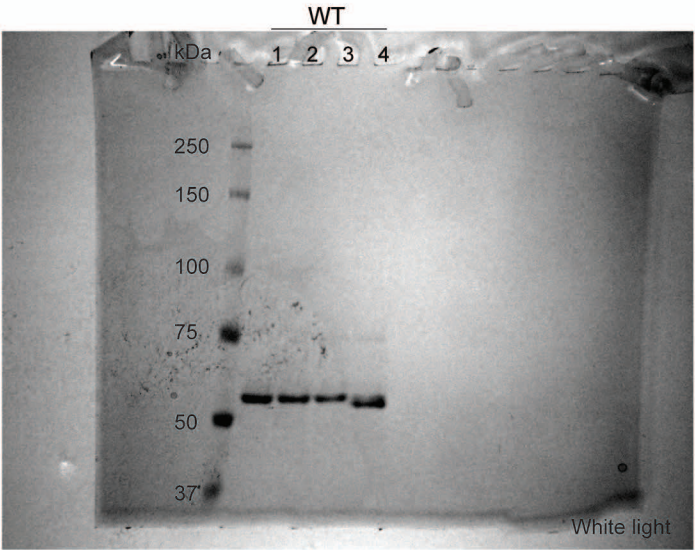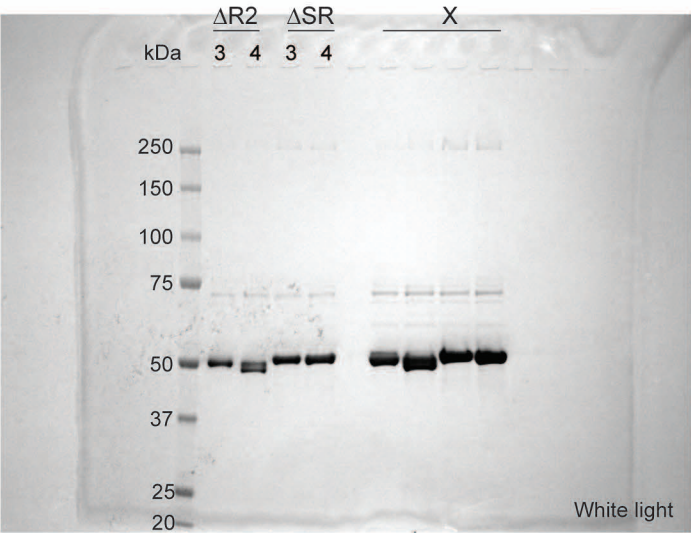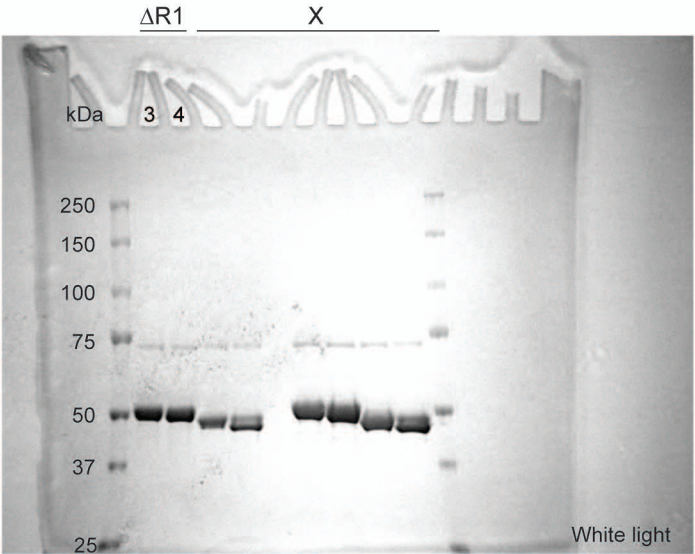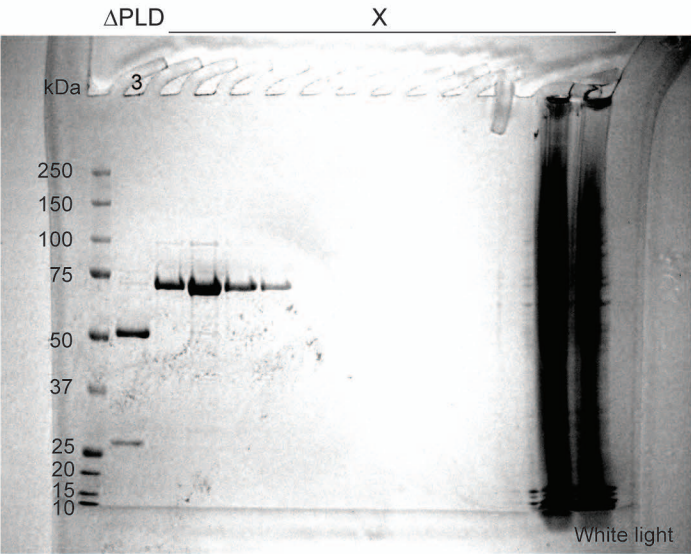

S8C Fig

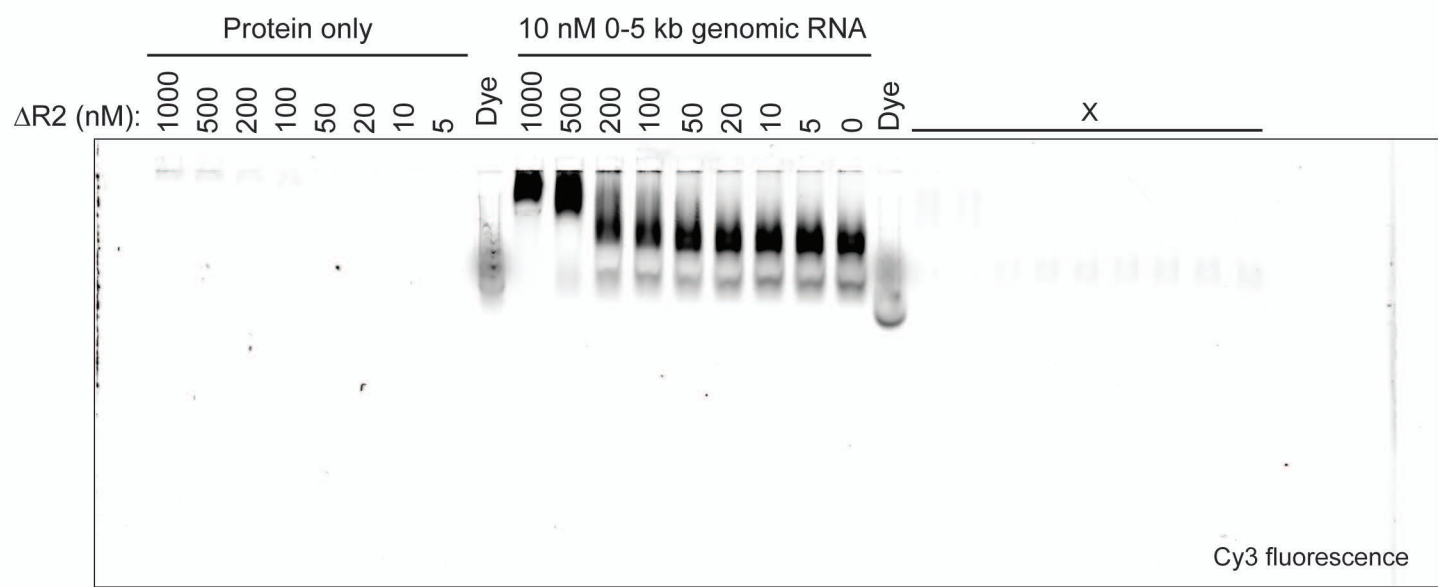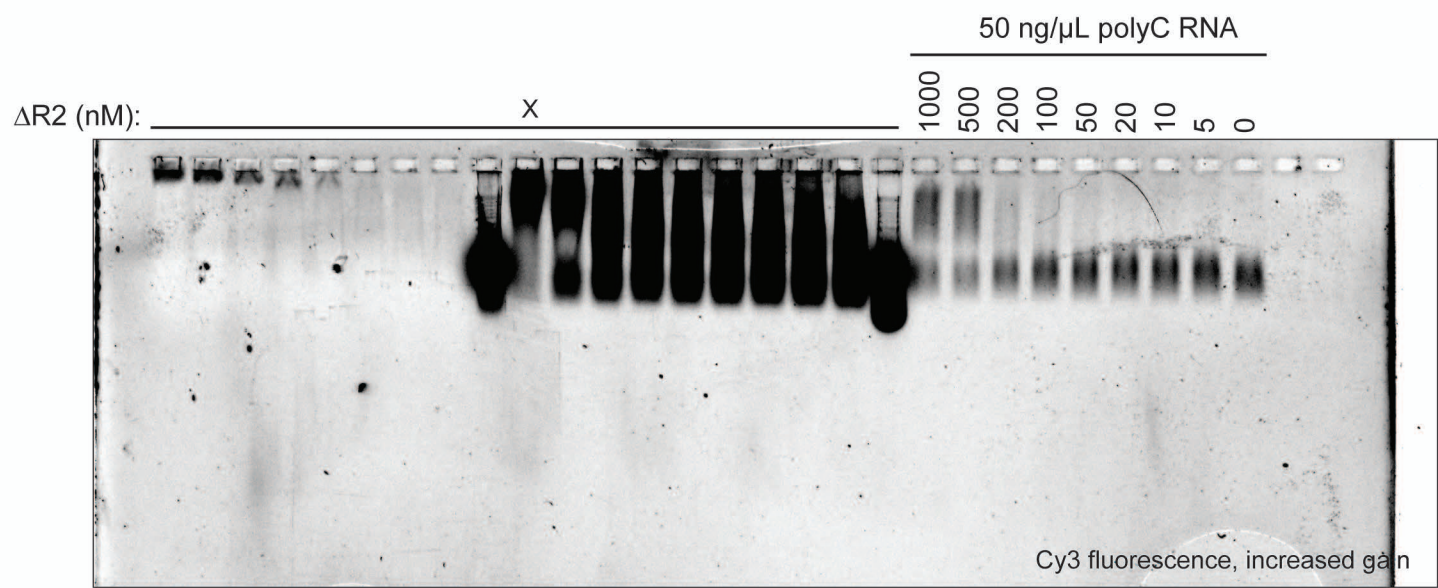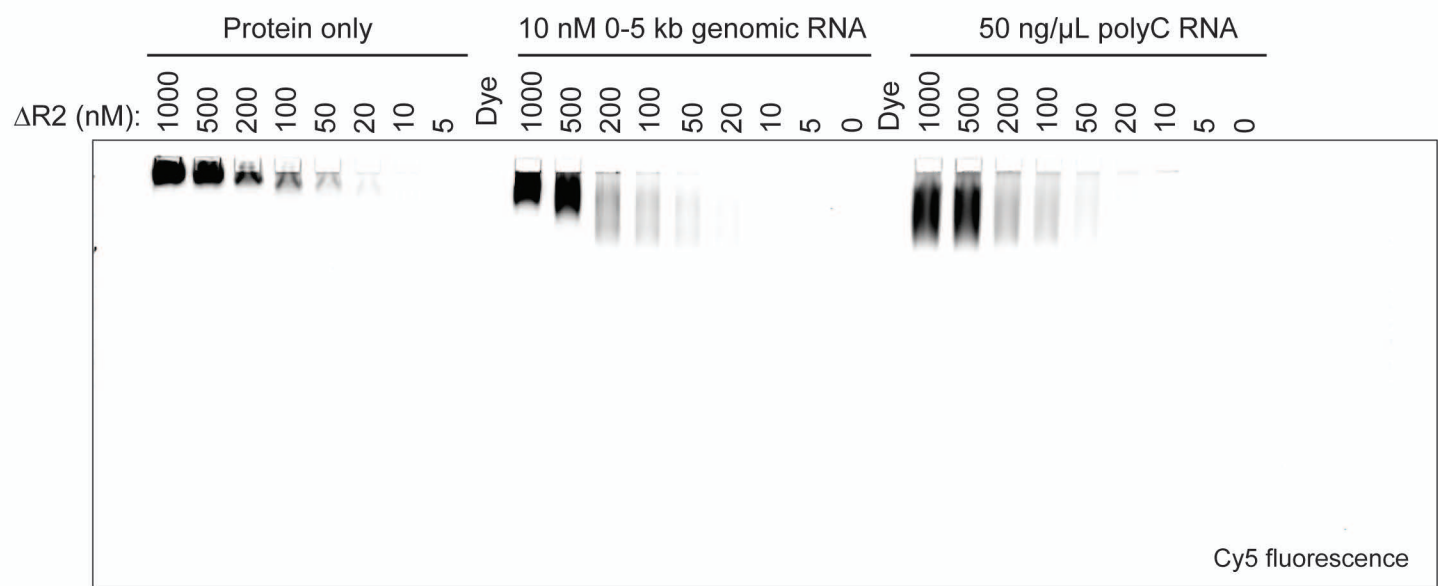

Supplement: S1 Raw Images — (PDF) [file pbio.3001425.s021.pdf]
