# Supplementary material for: SARS-CoV-2 nucleocapsid protein forms condensates with viral genomic RNA
Source: PLoS Biol. 2021 Oct 11;19(10):e3001425. doi: 10.1371/journal.pbio.3001425 (PMC8553124; doi:10.1371/journal.pbio.3001425)
Supplement: S1 Table — The cross-link (xlink) position indicates the amino acid number of the N protein sequence. The K169-K65 peptide is phosphorylated at S176. N, nucleocapsid. (DOCX) [file pbio.3001425.s016.docx]

| **Fold change** **(log_2_)** | **p-value** | **Xlink position 1** | **Xlink position 2** |
| --- | --- | --- | --- |
| -2.441 | 1.05E-04 | 257 | 266 |
| -2.226 | 5.88E-06 | 169 | 61 |
| -2.026 | 8.84E-08 | 266 | 347 |
| -1.697 | 1.23E-09 | 169 | 65 |
| -1.679 | 1.32E-09 | 169 (phosphoS176) | 65 |
| -1.409 | 1.21E-05 | 38 | 61 |
| -1.324 | 3.20E-05 | 169 | 266 |
| -1.079 | 6.84E-04 | 169 | 38 |
| -0.941 | 3.61E-12 | 266 | 299 |
| -0.796 | 8.00E-07 | 102 | 61 |
| -0.765 | 2.94E-06 | 266 | 266 |
| -0.762 | 3.87E-04 | 342 | 38 |
| -0.730 | 5.18E-07 | 233 | 266 |
| -0.729 | 8.10E-05 | 266 | 38 |
| -0.715 | 0.0034 | 355 | 375 |
| -0.648 | 0.0086 | 102 | 266 |
| -0.607 | 0.0013 | 342 | 355 |
| -0.594 | 2.84E-05 | 347 | 38 |
| -0.488 | 0.030 | 102 | 38 |
| -0.450 | 8.76E-04 | 266 | 375 |
| -0.448 | 1.53E-13 | 299 | 355 |
| -0.415 | 3.30E-06 | 233 | 38 |
| -0.382 | 0.048 | 38 | 65 |
| -0.380 | 2.21E-04 | 237 | 266 |
| -0.351 | 4.41E-04 | 266 | 388 |
| -0.277 | 0.037 | 266 | 374 |
| -0.264 | 2.72E-04 | 233 | 233 |
| -0.249 | 0.0043 | 375 | 38 |
| -0.222 | 0.015 | 233 | 248 |
| -0.220 | 0.034 | 347 | 388 |
| -0.185 | 0.042 | 342 | 388 |
| 0.103 | 0.038 | 233 | 249 |
| 0.271 | 0.0026 | 370 | 375 |
| 0.291 | 6.17E-06 | 233 | 237 |
| 0.347 | 6.53E-05 | 373 | 375 |
| 0.361 | 3.17E-06 | 237 | 388 |
| 0.378 | 5.90E-09 | 248 | 256 |
| 0.396 | 5.40E-05 | 256 | 342 |
| 0.398 | 1.41E-05 | 266 | 373 |
| 0.402 | 1.40E-06 | 257 | 342 |
| 0.402 | 9.55E-12 | 372 | 375 |
| 0.403 | 0.0014 | 237 | 249 |
| 0.408 | 1.05E-07 | 248 | 257 |
| 0.418 | 0.0024 | 370 | 374 |
| 0.428 | 3.83E-07 | 372 | 374 |
| 0.455 | 1.34E-06 | 266 | 372 |
| 0.522 | 1.41E-07 | 256 | 375 |
| 0.540 | 5.63E-06 | 256 | 38 |
| 0.578 | 3.49E-05 | 266 | 370 |
| 0.578 | 2.94E-05 | 257 | 374 |
| 0.580 | 4.27E-10 | 257 | 375 |
| 0.610 | 1.18E-07 | 237 | 257 |
| 0.641 | 1.58E-07 | 237 | 256 |
| 0.668 | 4.45E-04 | 248 | 388 |
| 0.670 | 4.93E-06 | 256 | 374 |
| 0.857 | 3.80E-12 | 237 | 248 |
| 0.902 | 1.31E-06 | 372 | 388 |
| 0.909 | 0.018 | 370 | 388 |
| 0.941 | 2.54E-05 | 248 | 249 |
| 0.976 | 2.58E-06 | 373 | 388 |
