# Supplementary material for: SARS-CoV-2 nucleocapsid protein forms condensates with viral genomic RNA
Source: PLoS Biol. 2021 Oct 11;19(10):e3001425. doi: 10.1371/journal.pbio.3001425 (PMC8553124; doi:10.1371/journal.pbio.3001425)
Supplement: S2 Table — (A) The coverage map and phosphorylation sites for the N protein detected in proteomics experiments. Text in red indicates peptide regions that were not detected; green highlight indicates an unambiguous phosphorylation site; and yellow highlight indicates an ambiguous phosphorylation site. (B) Each site identified by our study is characterized as being unambiguously or ambiguously localized based on manual inspection of the product ion series. See Methods for the full protein sequence and a link to supporting evidence. S176 is a phosphorylation site identified on a cross-linked peptide. MS, mass spectrometry; N, nucleocapsid. (DOCX) [file pbio.3001425.s017.docx]

**A**

MSDNGPQNQR NAPRITFGGP SDSTGSNQNG ERSGARSKQR RPQGLPNNTA SWFTALTQHG KEDLKFPRGQ

GVPINTNSSP DDQIGYYRRA TRRIRGGDGK MKDLSPRWYF YYLGTGPEAG LPYGANKDGI IWVATEGALN

TPKDHIGTRN PANNAAIVLQ LPQGTTLPKG FYAEGSRGGS QASSRSSSRS RNSSRNSTPG SSRGTSPARM

AGNGGDAALA LLLLDRLNQL ESKMSGKGQQ QQGQTVTKKS AAEASKKPRQ KRTATKAYNV TQAFGRRGPE

QTQGNFGDQE LIRQGTDYKH WPQIAQFAPS ASAFFGMSRI GMEVTPSGTW LTYTGAIKLD DKDPNFKDQV

ILLNKHIDAY KTFPPTEPKK DKKKKADETQ ALPQRQKKQQ TVTLLPAADL DDFSKQLQQS MSSADSTQAL

EGGGGWSHPQ FEKGGGSGGG SGGGSWSHPQ FEK

**B**

| **Phosphosite** | **Found in** | **Site Localization (this study)** |
| --- | --- | --- |
| S21 | This study | Unambiguous |
| S23 | This study, | Unambiguous |
| T24 | This study, | Unambiguous |
| S26 | This study, [1] | Ambiguous (S26\|S33) |
| S33 | This study | Ambiguous (S26\|S33) |
| T76 | [1, 2] |  |
| S78 | This study, [2] | Ambiguous (S78\|S79) |
| S79 | This study, [1, 2] | Unambiguous |
| S105 | [1, 2] |  |
| T141 | [2] |  |
| T166 | [2] |  |
| S176 | This study, [1, 2] | Unambiguous |
| S180 | [1, 2] |  |
| S183 | [1, 2] |  |
| S184 | [1, 2] |  |
| S194 | [1, 2] |  |
| S197 | [1] |  |
| T198 | [1, 2] |  |
| S201 | [1, 2] |  |
| S202 | [1, 2] |  |
| T205 | [1, 2] |  |
| S206 | [1, 2] |  |
| T391 | [2] |  |
| T393 | [3] |  |
| S412 | This study | Ambiguous (S412\|S413) |
| S413 | This study | Ambiguous (S412\|S413) |
| S441 | This study | Unambiguous |

**References**

1. Davidson AD, Williamson MK, Lewis S, Shoemark D, Carroll MW, Heesom KJ, et al. Characterisation of the transcriptome and proteome of SARS-CoV-2 reveals a cell passage induced in-frame deletion of the furin-like cleavage site from the spike glycoprotein. Genome Medicine. 2020;12(68).

2. Bouhaddou M, Memon D, Meyer B, White KM, Rezelj VV, Correa Marrero M, et al. The global phosphorylation landscape of SARS-CoV-2 infection. Cell. 2020;182(3):685-712.

3. Supekar NT, Shajahan A, Gleinich AS, Rouhani D, Heiss C, Azadi P. SARS-CoV-2 Nucleocapsid protein is decorated with multiple N- and O-glycans. bioRxiv. 2020; doi: 10.1101/2020.08.26.269043.
