# Supplementary material for: SARS-CoV-2 nucleocapsid protein forms condensates with viral genomic RNA
Source: PLoS Biol. 2021 Oct 11;19(10):e3001425. doi: 10.1371/journal.pbio.3001425 (PMC8553124; doi:10.1371/journal.pbio.3001425)
Supplement: S3 Table — (A) The proteins were ranked by SAF. These protein sequences were included in the CLMS search. Asterisks indicate exogenous proteins. (B) Cross-links between N and the G3BP1 and G3BP2 stress granule proteins identified in the qualitative CLMS experiment ranked by an SVM score. Values are frequency of detection (2 conditions with 2 replicates each). Xlink position indicates the amino acid number of the given protein sequence. SVM indicates the confidence that the cross-link is correctly identified. CLMS, cross-linking mass spectrometry; MS, mass spectrometry; N, nucleocapsid; SAF, spectral abundance factor; SVM, support vector machine. (DOCX) [file pbio.3001425.s018.docx]

**A**

| **Gene** | **Unique Peptides** | **Percent Coverage** | **SAF** | **Species** | **UniProt Accession** | **Protein Name** |
| --- | --- | --- | --- | --- | --- | --- |
| N | 57 | 70.0 | 104.6 | SARS2 | P0DTC9 | Nucleoprotein |
| HSPA1A | 54 | 59.3 | 27.5 | HUMAN | P0DMV8 | Heat shock 70 kDa protein 1A |
| G3BP2 | 38 | 42.5 | 19.5 | HUMAN | Q9UN86 | Ras GTPase-activating protein-binding protein 2 |
| G3BP1 | 37 | 60.5 | 17.8 | HUMAN | Q13283 | Ras GTPase-activating protein-binding protein 1 |
| HSPA8 | 27 | 37.8 | 9.8 | HUMAN | P11142 | Heat shock cognate 71 kDa protein |
| RPS12 | 2 | 17.4 | 8.3 | HUMAN | P25398 | 40S ribosomal protein S12 |
| NA | 4 | 17.3 | 7.4 | PIG* | P00761 | Trypsin |
| TUBB | 20 | 56.3 | 7.0 | HUMAN | P07437 | Tubulin beta chain |
| TUBA1B | 19 | 54.3 | 6.7 | HUMAN | P68363 | Tubulin alpha-1B chain |
| TUBB4B | 18 | 50.6 | 6.5 | HUMAN | P68371 | Tubulin beta-4B chain |
| KRT9 | 14 | 52.6 | 6.1 | HUMAN | P35527 | Keratin, type I cytoskeletal 9 |
| A2M | 27 | 20.7 | 5.8 | BOVIN* | Q7SIH1 | Alpha-2-macroglobulin |
| HSPA5 | 17 | 29.5 | 5.2 | HUMAN | P11021 | Endoplasmic reticulum chaperone BiP |
| NPM1 | 6 | 37.1 | 5.1 | HUMAN | P06748 | Nucleophosmin |

**B**

| **Protein 1** | **Xlink position 1** | **Protein 2** | **Xlink position 2** | **SVM score** |
| --- | --- | --- | --- | --- |
| G3BP2_HUMAN | 50 | G3BP2_HUMAN | 281 | 2.17 |
| Nucleoprotein | 375 | G3BP1_HUMAN | 453 | 1.63 |
| G3BP2_HUMAN | 50 | Nucleoprotein | 266 | 1.56 |
| G3BP2_HUMAN | 50 | Nucleoprotein | 102 | 1.26 |
| G3BP2_HUMAN | 370 | G3BP2_HUMAN | 407 | 1.04 |
| G3BP2_HUMAN | 365 | G3BP2_HUMAN | 407 | 0.88 |
| G3BP2_HUMAN | 50 | Nucleoprotein | 38 | 0.71 |
| Nucleoprotein | 388 | G3BP1_HUMAN | 453 | 0.67 |
| Nucleoprotein | 266 | G3BP1_HUMAN | 393 | 0.14 |
| Nucleoprotein | 374 | G3BP1_HUMAN | 453 | 0.00 |
