# Supplementary material for: SARS-CoV-2 nucleocapsid protein forms condensates with viral genomic RNA
Source: PLoS Biol. 2021 Oct 11;19(10):e3001425. doi: 10.1371/journal.pbio.3001425 (PMC8553124; doi:10.1371/journal.pbio.3001425)
Supplement: S4 Table — The parameters of fitting to a dose–response equation in the presence and absence of polyC RNA in response to increasing concentrations of salt or nelfinavir mesylate. N, nucleocapsid. (DOCX) [file pbio.3001425.s019.docx]

| **Condition** | **IC_50_ / EC_50_** | ***n*** | **R^2^** | **Fig** |
| --- | --- | --- | --- | --- |
| N with NaCl, condensate volume | 90 ± 1 mM | 12.26 ± 0.06 | 1 | 1D |
| N + PolyC with NaCl, condensate volume | 152 ± 6 mM | 11 ± 9 | 0.97 | 1D |
| N + PolyC with nelfinavir mesylate, condensate number | 4.3 ± 1.3 μM | 1.9 ± 1.0 | 0.90 | 5B |
